# A Role for the Chaperone Complex BAG3-HSPB8 in Actin Dynamics, Spindle Orientation and Proper Chromosome Segregation during Mitosis

**DOI:** 10.1371/journal.pgen.1005582

**Published:** 2015-10-23

**Authors:** Margit Fuchs, Carole Luthold, Solenn M. Guilbert, Alice Anaïs Varlet, Herman Lambert, Alexandra Jetté, Sabine Elowe, Jacques Landry, Josée N. Lavoie

**Affiliations:** 1 Centre de Recherche sur le Cancer de l’Université Laval and Département de Biologie Moléculaire, Biochimie Médicale et Pathologie, Université Laval, Quebec, Canada; 2 Oncology, Centre de Recherche du CHU de Québec-Université Laval, Quebec, Canada; 3 Reproduction, Perinatal Health and Child Health, Centre de Recherche du CHU de Québec-Université Laval, Quebec, Canada; 4 Centre de Recherche sur le Cancer de l’Université Laval and Département de Pédiatrie, Université Laval, Quebec, Canada; The University of Arizona, UNITED STATES

## Abstract

The co-chaperone BAG3, in complex with the heat shock protein HSPB8, plays a role in protein quality control during mechanical strain. It is part of a multichaperone complex that senses damaged cytoskeletal proteins and orchestrates their seclusion and/or degradation by selective autophagy. Here we describe a novel role for the BAG3-HSPB8 complex in mitosis, a process involving profound changes in cell tension homeostasis. BAG3 is hyperphosphorylated at mitotic entry and localizes to centrosomal regions. BAG3 regulates, in an HSPB8-dependent manner, the timely congression of chromosomes to the metaphase plate by influencing the three-dimensional positioning of the mitotic spindle. Depletion of BAG3 caused defects in cell rounding at metaphase and dramatic blebbing of the cortex associated with abnormal spindle rotations. Similar defects were observed upon silencing of the autophagic receptor p62/SQSTM1 that contributes to BAG3-mediated selective autophagy pathway. Mitotic cells depleted of BAG3, HSPB8 or p62/SQSTM1 exhibited disorganized actin-rich retraction fibres, which are proposed to guide spindle orientation. Proper spindle positioning was rescued in BAG3-depleted cells upon addition of the lectin concanavalin A, which restores cortex rigidity. Together, our findings suggest the existence of a so-far unrecognized quality control mechanism involving BAG3, HSPB8 and p62/SQSTM1 for accurate remodelling of actin-based mitotic structures that guide spindle orientation.

## Author Summary

Small heat shock proteins (sHSP/HSPB) form a diverse family of ATP-independent chaperones. Some of them protect the proteome against aggregation during stress and others regulate normal biological processes through ill-defined mechanisms. Interactions between HSPB proteins and elements of the cytoskeleton are increasingly linked to their implication in human degenerative diseases and cancer. For instance, a multichaperone complex containing HSPB8 and its co-chaperone BAG3 would maintain muscle cell integrity by promoting the autophagic clearance of damaged components within F-actin structures. Selective autophagy is a targeted protein degradation mechanism for elimination of damaged organelles and proteins. It may also regulate removal of signaling proteins from their functionally relevant sites during intense remodeling of the cytoskeleton, as it occurs during mitosis. Here, we report a novel role for HSPB8 and BAG3 during mitosis in mammalian cells that involves the autophagic receptor p62/SQSTM1. We show that a reduction of any protein within the HSPB8-BAG3-p62/SQSTM signaling axis similarly impairs mitotic progression and chromosome segregation by affecting orientation of the mitotic spindle and assembly of mitotic-specific actin structures. Our findings establish a unique role for HSPB8 in a novel function of BAG3 in mitotic cell division and genome stability, through effect on remodeling of the actin cytoskeleton.

## Introduction

Heat shock proteins (HSP) are molecular chaperones with key roles within the so-called proteostasis network. This network is composed of elaborate pathways that allow cells to protect their proteome from aggregation, facilitate the assembly of multi-components complexes, and maintain the integrity of cytoskeleton polymers by eliminating damaged components in response to a variety of stress [1, 2]. As molecular chaperones, HSP detect misfolded proteins and facilitate their refolding, seclusion or degradation. They provide molecular connections with the ubiquitin-proteasome system and the macroautophagy machinery (hereafter named autophagy). In addition, associations of HSP with co-chaperones allow them to be recruited to specific, yet unrelated biological processes [3]. These processes nevertheless share a requirement for dynamic assembly-disassembly of multiprotein complexes at a given location and time, which often involve protein conformational changes. HSP, not surprisingly, are believed to support the phenotype of tumor cells in several ways, mostly as guardians of the proteome against aggregation [4]. Indeed, a proteotoxic stress response typified by upregulation of HSP is proposed to characterize most human malignant cells that experience increased proteomic instability [5].

The small heat shock proteins (HSPB) form a diverse and enigmatic family of chaperones, for which there is currently no single model of mechanism of action [6, 7]. They are viewed as proteins able to confer protection against the deleterious effect of stresses by virtue of their strong induction after stress [8]. Many of them have been shown to act as ATPase-independent holdases to prevent protein aggregation. Noncanonical functions have also been uncovered for ubiquitously expressed HSPB proteins in signaling, with an increasingly recognized connection between HSPB proteins and cytoskeleton elements. We, and others, have shown that the prototypal member of this family heat shock protein B1 (HSPB1/HSP27) performs specialized function in the regulation of actin filaments architecture during both physiological and pathological stimulations [2, 9, 10]. Consistent with this, HSPB1 has been involved in several processes requiring extensive actin polymerization, including tumor cell migration and invasion [8]. Likewise, heat shock protein B8 (HSPB8/HSP22) forms a stable complex with the co-chaperone BCL2-associated athanogene 3 (BAG3) that has emerged as a key element of cytoskeletal proteostasis in muscle cells [11, 12]. Little is known, however, of the function of this chaperone complex in highly dividing tumor cells where BAG3 has been linked to cytoskeletal dynamics. This is of particular interest given that expression of BAG3 is upregulated in several primary tumors and tumor cell lines of various origins, where it is believed to support the tumor phenotype [13].

BAG3 is one of a family of co-chaperones characterized by a C-terminal BAG domain that binds the HSP70/HSPA ATPase domain to regulate the fate of HSP70 substrates [14]. We have identified BAG3 as an obligate partner of HSPB8 in several cell lines where the depletion of BAG3 leads to a rapid degradation of HSPB8 [15]. Still, the significance of its strong connection to HSPB8 has remained unclear, as BAG3 can function independently of HSPB8 in protein quality control. BAG3 in association with HSP70/HSC70 has been implicated in the aggresome-autophagy pathways. When proteasome-dependent degradation fails, BAG3 would promote the sequestration and targeting of HSP70/HSC70-associated protein aggregates to the aggresome, a perinuclear compartment with high autophagic activity [16–19] While overexpression of HSPB8 can stimulate autophagy in a BAG3-dependent manner, it seems to be dispensable for the function of BAG3 in the aggresome-autophagy pathways during proteotoxic stress [15, 18, 20].

Recent work suggests that the BAG3-HSPB8 complex could play more specialized function in cytoskeletal proteostasis. In the context of muscle cells, BAG3 and its associated chaperones regulate the structural integrity of the sarcomere—the actin-contractile structure of myofibrils, through effects on localization, stabilization, or clearance of essential actin-binding components [21–23]. Such function is considered to be crucial for maintenance of muscle cell integrity as mutations in BAG3 are associated with the development of muscle disease in children [24, 25]. This suggests that the connection between BAG3 and HSPB8 may serve in some capacity to recruit specialized chaperone systems to biological processes relying on high-order assembly-disassembly of actin. BAG3 is unique among the BAG proteins for the presence of a WW domain and a proline-rich region (PXXP), which might provide BAG3 with the capacity to act as a protein scaffold to integrate chaperone systems with signaling pathways controlling cell remodeling [26, 27]. Both motifs have been shown to contribute to the signaling effects of BAG3 on cell adhesion, actin assembly and motility of tumor cells [28–30]. Nevertheless, the relationship between BAG3-mediated effects on actin dynamics and HSPB8 in dividing cells remains largely unexplored.

We postulate that an HSPB8-dependent function of BAG3 could be mobilized during mitosis to assist in the proper and timely assembly of cytoskeletal structures. Mitosis involves the most dramatic and spectacular changes in the overall structure of a cell. The actin cytoskeleton undergoes extensive remodeling to allow for cell-shape changes that are required for proper transition into and out of mitosis [31]. Therefore, we investigated the relationship between BAG3 and HSPB8 expression, proper progression of cells through mitosis and mitotic reorganization of cellular architecture. We demonstrate the existence of a novel HSPB8-dependent mitotic function of BAG3 that would facilitate the timely remodeling of actin-based mitotic structures that guide spindle orientation and proper chromosome segregation.

## Results

### BAG3 exhibits mitotic-specific features

**Fig 1:**
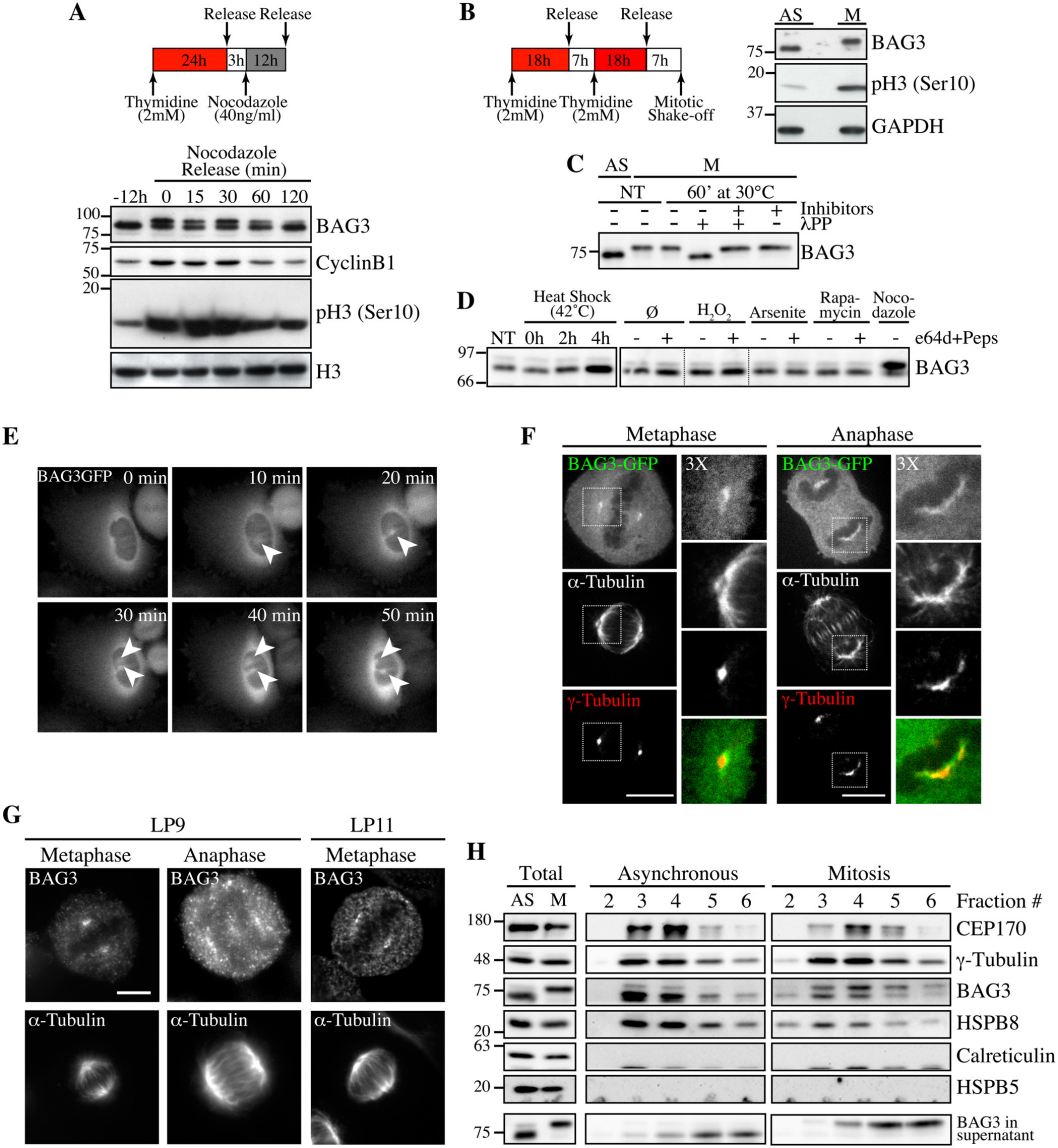
Hyperphosphorylation and centrosomal localization of BAG3 in mitotic cells. (A) HeLa cells were harvested at different times during release from a Thymidine/nocodazole block (time 0). Cell lysates were immunoblotted with the indicated antibodies. (B) Mitotic HeLa-GFP-H2B cells synchronized by the double Thymidine block method and recovered by mitotic shake off were lysed and processed for immunoblotting for BAG3, phospho-histone H3 (p-H3) and GAPDH as loading control; AS, asynchronous; M, mitotic. (C) BAG3 immunoprecipitates (IPs) were prepared from asynchronous HeLa cells (AS) or from HeLa cells arrested in mitosis by incubation with nocodazole for 16 h (M, 400 ng/ml). BAG3 IPs were left untreated (NT) or incubated at 30°C in the presence of phosphatase (lPP) with (+) or without (-) phosphatase inhibitors for 60 min and were analyzed by Western blot. (D) Western blot of 293T cell lysates that have been treated as followed: heat shock at 42°C for the indicated times; H_2_O_2_ (500 mM, 4 h); arsenite (200 mM, 4 h); rapamycin (200 nM, 4 h); nocodazole (400 ng/ml, 16 h), with (+) or without (-) inhibitors of lysosomal proteases (E64d, 10 μg/ml; pepstatin, 10 μg/ml). (E) HeLa-BAG3-GFP cells were imaged by time-lapse fluorescence microscopy; arrowheads designate the centrosomes and surrounding perinuclear material. (F) Confocal images of mitotic HeLa-BAG3-GFP cells synchronized by a 24 h-Thymidine block and released in fresh medium for 8 h, showing staining of a-tubulin and g-tubulin. (G) Confocal images of mitotic HeLa cells showing staining of a-tubulin and endogenous BAG3 with polyclonal anti-BAG3 antibodies (LP9, LP11); Bars, 10 μm. (H) Westerns blots of centrosomal fractions purified from asynchronous (AS) or from mitotic (M) HeLa cells synchronized with nocodazole; centrosomal markers: CEP170, g-tubulin; calreticulin and HSPB5: negative controls; Total: proteins levels in total cell extracts.

In the course of experiments examining BAG3 levels at different steps of the cell cycle, we observed that prolonged treatments of HeLa or 293T cells with nocodazole, a microtubule poison arresting cells in mitosis, induced a BAG3 supershifted band on SDS-PAGE (Fig 1A). The supershifted band appeared to vanish along with cyclin B1 degradation, suggesting that BAG3 was specifically modified in early mitosis (Fig 1A). Indeed when mitotic HeLa cells were recovered by mitotic shake off, the supershifted band of BAG3 accounted for nearly 100% of the BAG3 detected (Fig 1B, *M*). Significantly, treatment of mitotic cell extracts with lambda phosphatase (lPP) led to a disappearance of the supershifted band (Fig 1C). The BAG3 supershifted band was not observed in extracts from cells submitted to a variety of stress, including heat shock that rather increased BAG3 levels as expected (Fig 1D). These observations suggested that BAG3 was specifically hyperphosphorylated at mitotic entry.

Time-sequences from confocal microscopy showed that BAG3-GFP localized to centrosomes or the surrounding perinuclear material, as they separated before nuclear envelope breakdown (Fig 1E). At metaphase through anaphase, BAG3-green fluorescent protein (GFP) was found at spindle poles and showed overlapping distribution with the centrosomal marker g-tubulin in fixed cells (Fig 1F). Similarly, endogenous BAG3 was detected at spindle poles of HeLa cells at metaphase and anaphase, using two distinct anti-BAG3 antibodies (lapin 9 [LP9] and LP11; Fig 1G). In agreement with our immunolocalization analyses, BAG3 and its chaperone partner HSPB8 codistributed with the centrosomal markers g-tubulin and centrosome protein of 170 kDa (CEP170) in cellular fractions purified from both asynchronous (AS) and mitotic (M) HeLa cell extracts (Fig 1H, fractions 3–5). Intriguingly, the fast migrating, hypophosphorylated form of BAG3 was abundant in purified centrosomal fractions from mitotic cells, while in post-nuclear supernatants, the supershifted BAG3 band was predominant (Fig 1H, total). This suggested that mitotic-specific phosphorylation of BAG3 could change its localization and potentially its binding partners. The centrosomal enrichment of hypophosphorylated BAG3 was not due to protein dephosphorylation during the purification procedure, as the supershifted BAG3 protein could be recovered in the supernatant fraction (Fig 1H; BAG3 in supernatant). In contrast to HSPB8, HSPB5/aB-crystallin was not found in centrosomal fractions although it was clearly detected in total cell lysates (Fig 1H). The specific changes in phosphorylation of BAG3 and its codistribution with HSPB8 at the centrosome prompted us to investigate a role for the chaperone complex in early mitosis.

### BAG3 modulates the progression trough early mitosis

**Fig 2:**
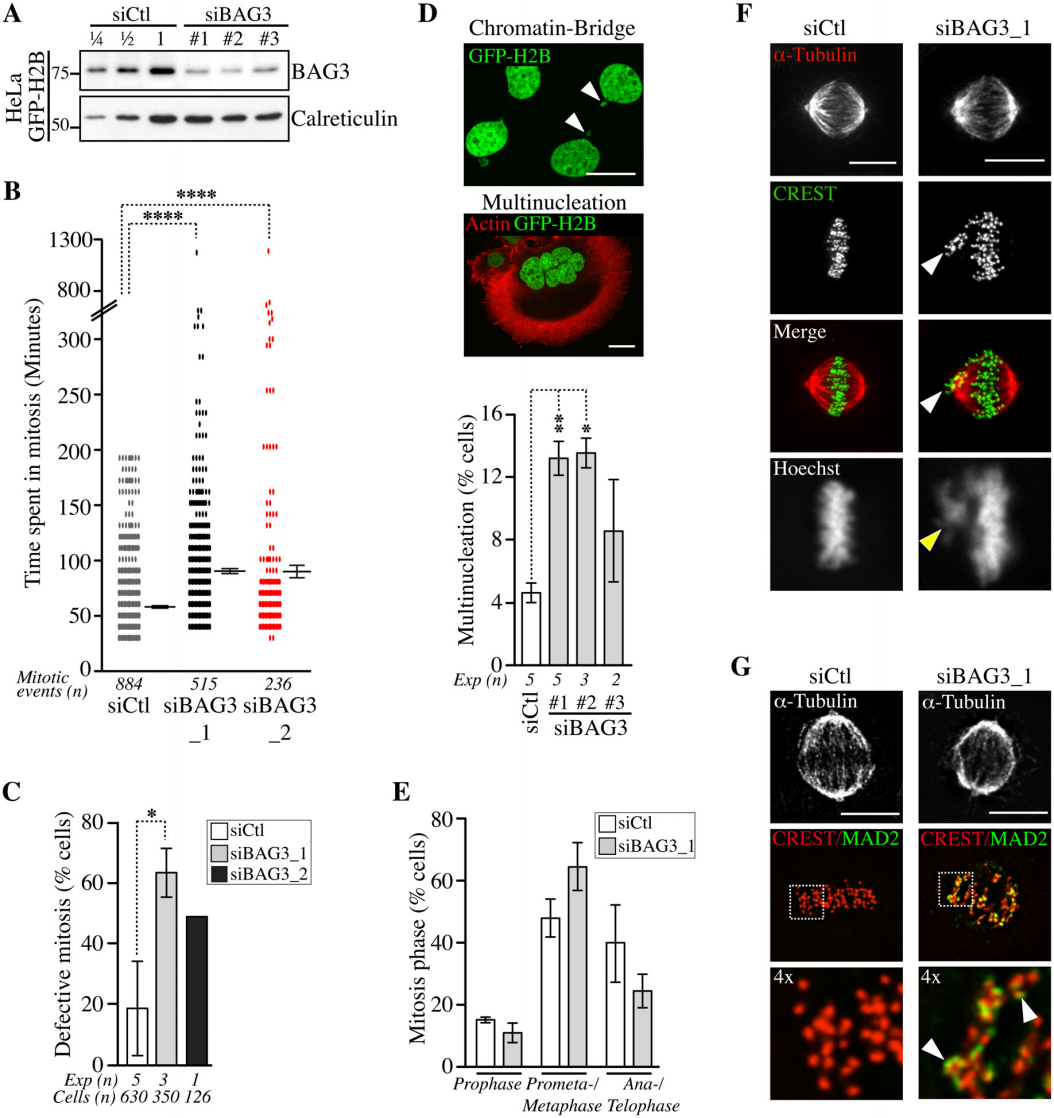
Depletion of BAG3 delays cells in mitosis and impairs chromosome segregation. (A) Representative Western blots of extracts of HeLa-GFP-H2B cells showing BAG3 depletion levels after transfection with different BAG3-specific siRNAs as indicated. Depletion was estimated at > 75% by loading increasing amounts of control extracts (siCtl); calreticulin levels: loading control. (B) Graph depicting the time spent in mitosis determined from time-lapse imaging of unsynchronized HeLa-GFP-H2B cells transfected with the indicated siRNAs. The means +/- SE are from 884 or 515 mitotic events from 4 or 3 independent experiments (siCtl, siBAG3_1, respectively), or from 236 mitotic events from 1 representative experiment (siBAG3_2) (S1 Dataset). (C) Graph showing the percentages of HeLa-GFP-H2B cells treated and imaged as in B, which exhibited typical mitotic defects: delay in prometaphase-metaphase, chromosome misalignment and multipolar spindle; means +/- SD calculated from individual cells (*n*) from independent experiments (*Exp*). (B, C) Mann-Whitney’s tests were performed relative to control, ****, *p* < 0.0001, *, *p* < 0.05. (D) Confocal images of HeLa-GFP-H2B cells transfected with BAG3-specific siRNAs, showing chromatin bridge (white arrowheads) or multinucleation; green: GFP-H2B; red: F-actin staining; Bars, 20 μm. The graph shows the incidence of multinucleation, which was estimated 72 h after transfection from 2 to 5 independent experiments as indicated; n>650 cells; **, p < 0.01; *, p < 0.05 (Mann-Whitney test). (E-G) siRNA-treated HeLa-GFP-H2B cells were synchronized in mitosis by the double Thymidine block method and processed for staining of kinetochores (CREST staining), spindle microtubules (a-tubulin staining) and SAC protein (MAD2 staining). The graph depicts percentages of cells at different stages of mitosis; means +/- SD from >500 cells from 3 independent experiments. ****, p < 0.0001, compare siBAG3 to siCtl (prometaphase-metaphase cells) by the Exact Fisher Test. Confocal image stacks show BAG3-depleted cells with characteristic unaligned kinetochores (arrowheads in F) and strong MAD2 staining emphasized in enlarged views of the boxed regions (arrowheads in G); Bars, 10 μm.

To explore a role for BAG3 in mitosis, we first assessed the impact of BAG3 depletion on the progression of unperturbed mitotic events using HeLa cells, which are widely used for mitotic studies. Unsynchronized HeLa cells stably expressing a marker of chromatin GFP-histone 2B (HeLa-GFP-H2B) were transfected with control small interfering RNA (siRNAs), or BAG3-specific siRNAs that achieved >75% depletion of BAG3 in these cells (Fig 2A). Cells were imaged for a period of 48 h to 72 h starting at 40 h after transfection to track the genealogy of cells and establish a mitotic pedigree for families of daughter cells (S1A Fig). By doing so, we found that BAG3 depletion markedly reduced the incidence of cell division. This was reflected by a >5-fold increase in the proportion of cells that divided only once upon depletion of BAG3 relative to control cells (~ 33% compared to ~ 6%; n> 64 cells; first 48 h). Consistently, the mean time required to complete a mitotic event, as determined from nuclear envelope breakdown to anaphase onset, was significantly increased in BAG3-depleted cells (~90.33 +/- 3.15 min; n = 515 mitotic events for siBAG3_1; ~90.29 +/- 8.28 min n = 236 mitotic events for siBAG3_2) compared to control cells (~58.90 +/- 1.33 min; n = 884 mitotic events) (Fig 2B). The delay in mitosis was particularly severe in sub-populations of BAG3-depleted cells, which spent between 5 h to 20 h in mitosis. Overall, there was a 2.6- to 3.4-fold increase in the proportion of BAG3-depleted cells that exhibited defective mitosis mainly characterized by an increase in the time spent in mitosis (Fig 2C). While BAG3-depleted cells eventually progressed through anaphase, the incidence of nuclear abnormalities resulting from aberrant mitosis and cytokinesis (i.e. micronucleation and multinucleation) was substantially increased (Fig 2D). Micronucleated and multinucleated cells often result from chromosomes that have not been properly captured and lag behind during anaphase forming an anaphase bridge (Fig 2D, arrowheads) [32]. Similar nuclear abnormalities were also observed in MCF7 cells transfected with BAG3-specific siRNAs, indicating that the effects of BAG3 on cell division are not cell type-specific (S1B and S1C Fig).

We further characterized the mitotic delay caused by depletion of BAG3 by scoring cells at different stages of mitosis after staining of kinetochores (CREST staining) and spindle microtubules (a-tubulin staining) (S6 Fig). There was a significant increase in the proportion of BAG3-depleted cells at prometaphase, or at metaphase with unaligned chromosomes at the spindle poles (Fig 2E and 2F, arrowheads). Immunofluorescence using anti-mitotic arrest-deficient gene 2 (MAD2) antibody showed that this essential spindle checkpoint (SAC) protein was present at unaligned kinetochores in BAG3-depleted cells (Fig 2G) [33]. Together with the mitotic delay observed, these results suggest that BAG3 depletion does not perturb the function of the SAC.

### BAG3 depletion impairs the three-dimensional positioning of the spindle

**Fig 3:**
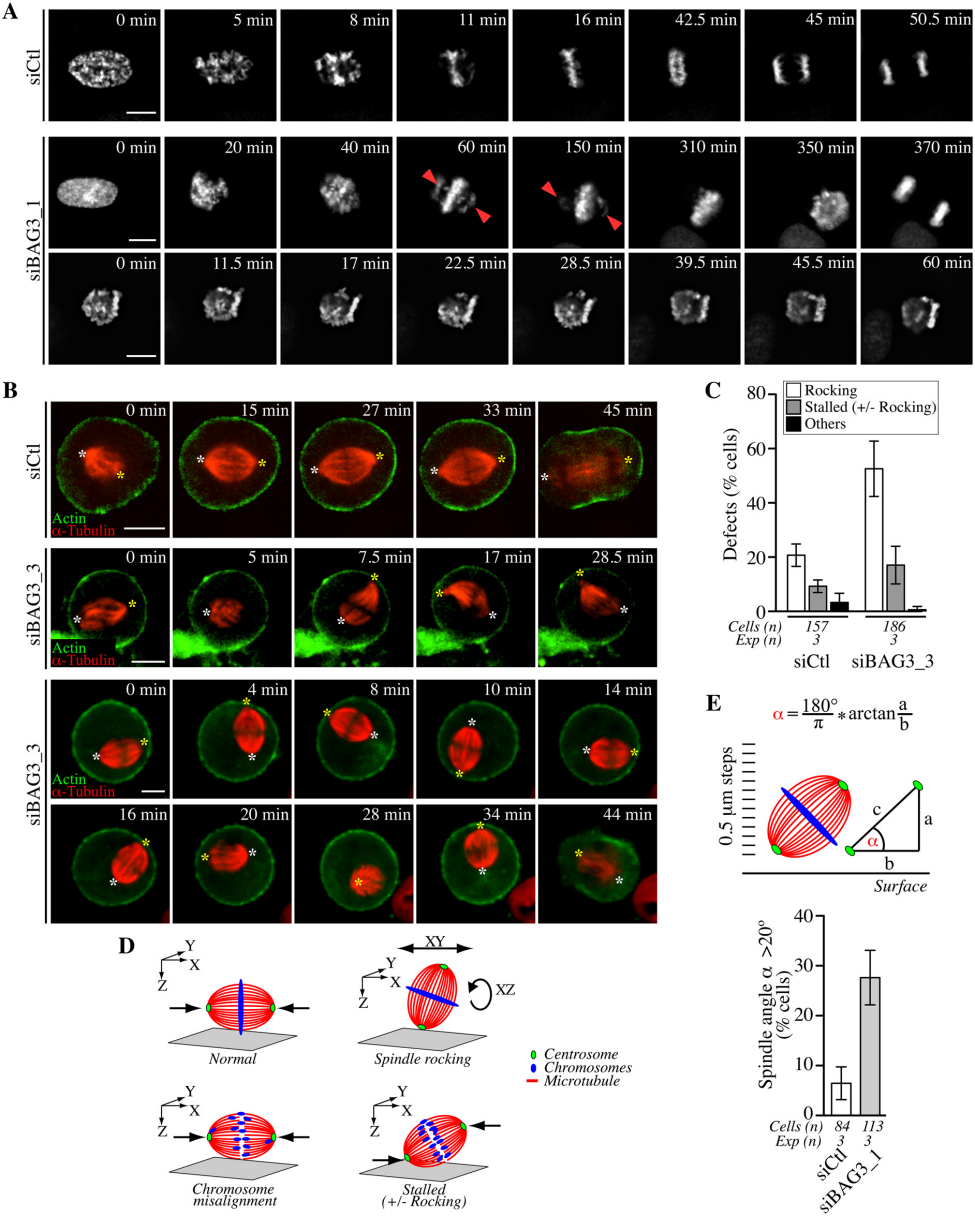
Bag3 regulates chromosome congression and spindle orientation. (A) Deconvolved time-lapse sequences of siRNA-treated HeLa-GFP-H2B cells; siCTL: control siRNA; siBAG3_1: BAG3-specific siRNA; arrowheads designate unaligned chromosomes remaining at spindle poles for prolonged periods. (B) Confocal time-lapse sequences of siRNA-treated HeLa-RFP-H2B expressing low, non-interfering levels of RFP-a-tubulin and GFP-actin. Cells were synchronized by the double Thymidine block method and were imaged for a 1 h-period at ~1.5 min intervals. Asterisks designate spindle poles that remained stable from prometaphase to anaphase onset in control cells (siCtl; S1 Movie), but displayed abnormal movements in BAG3-depleted cells (siBAG3_3; S2 and S3 Movies). (C) Quantification of cells treated as in A, depicting abnormal spindle dynamics: rocking, large movements in the *xy* plane and/or rotations in the *z*-axis; stalled +/- rocking, arrested in prometaphase or metaphase for > 30 min; means +/- SD from >150 cells from 3 independent experiments (S1 Dataset). (D) Schematic of the mitotic phenotypes associated with BAG3 depletion; green: centrosomes; red: spindle microtubules; blue: chromosomes. (E) Scheme of the method used to measure the spindle angle *a* in fixed cells. siRNAs-treated HeLa-GFP-H2B cells were synchronized by the double Thymidine block method and processed for IF with anti-a-tubulin, anti-g-tubulin and Hoechst. The graph shows percentages of cells that exhibited a spindle angle *a* >20°; means +/- SE from >84 cells from 3 independent experiments; **, p = 0.0072, compare siBAG3 to siCtl by the Exact Fisher Test.

To further define the effect of BAG3 depletion on the process of chromosome congression to the metaphase plate in live cells, we imaged siRNA-treated HeLa-GFP-H2B cells that have been synchronized in mitosis, by spinning disk confocal microscopy. Live-cell imaging in HeLa-GFP-H2B recapitulated the mitotic delay observed in Fig 2B and demonstrated that this delay is associated with two main phenotypes (Fig 3A, siBAG3): 1- Some BAG3-depleted cells were attempting to align chromosomes that remained localized at spindle poles for prolonged period of time (>60 min), before finally progressing through anaphase (Fig 3A, second row of panels); 2- Others appeared to be stalled at the prometaphase stage (>60–90 min) with chromosomes dispersed in different focal planes (Fig 3A, third row of panels). Thus live cell imaging data were consistent with a delay in satisfying the spindle assembly checkpoint caused by chromosome congression defects upon reduction of BAG3 levels.

We reasoned that improper alignments of chromosomes that often looked like donuts in BAG3 depleted cells at the metaphase stage (Fig 3, “350 min” in second row) could result in part from incorrect positioning of the mitotic spindle, which is normally oriented parallel to the surface of the coverslip in nonpolarized cells [34]. To then analyze the effect of BAG3 depletion on mitotic spindle behavior, we imaged siRNA-transfected HeLa cells stably expressing red fluorescent protein (RFP)-H2B after having delivered in these cells low, non-interfering levels of RFP-a-tubulin and GFP-actin. Cells that have been transfected with control siRNAs rapidly aligned their mitotic spindle parallel to the substrate (Fig 3B, siCtl; S1 Movie). In marked contrast, time-lapse sequences revealed that mitotic spindles were unusually motile and incorrectly positioned within cells upon depletion of BAG3 (Fig 3B, siBAG3; S2 and S3 Movies). In a significant proportion of BAG3-depleted cells (~52.8%), spindles frequently rotated in the *z*-axis and showed large movements in the *xy* plane, while aberrant spindle rocking was observed in only ~20% of cells transfected with control siRNAs (Fig 3C and 3D).

To quantify the spindle mispositioning defects, we measured the spindle angle *a* relative to the surface of the coverslip in fixed cells. HeLa-GFP-H2B cells were transfected with siRNAs and stained with anti-a-tubulin and anti-g-tubulin after cell synchronization. In control cells at metaphase, the average spindle angle was ~8.3° +/- 0.6, in agreement with previous work [35, 36]. A marginal, but significant increase in the average spindle angle was measured in cells at metaphase upon depletion of BAG3 (~12.6° +/- 0.8). Under these conditions, however, there was a remarkable 4-fold increase in the proportion of BAG3-depleted cells (~ 27.7% +/- 9.5) relative to control cells (~ 6.6% +/- 5.6) showing a spindle angle of >20° more typical of cells at the prometaphase stage that have not completed alignment (Fig 3E) [36]. These data confirmed that a significant proportion of BAG3-depleted cells underwent *z*-axis spindle rotations, which could delay chromosome congression and anaphase onset. Indeed, there was higher proportion of BAG3-depleted cells showing mitotic delays (>30 min in prometaphase-metaphase) along with spindle rocking in time-lapse analyses (Fig 3C).

### The effect of BAG3 on mitotic spindle dynamics involves its chaperone partner HSPB8, but not HSP70/HSC70

**Fig 4:**
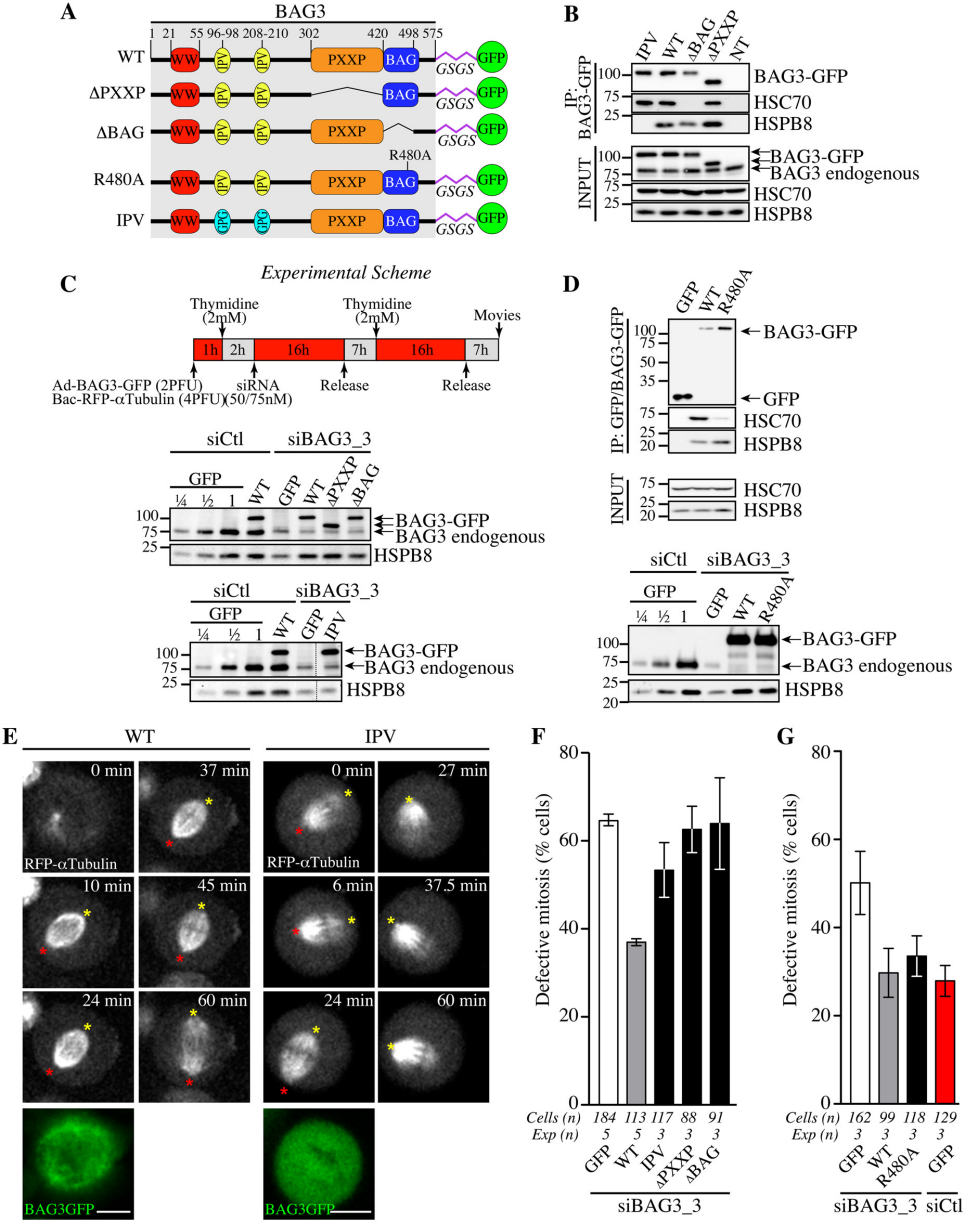
Structural requirements on BAG3 for restoration of normal spindle dynamics in BAG3-depleted cells. (A) Schematic of BAG3-GFP proteins bearing mutations or deletions of functional domains and a glycine/serine-rich flexible linker peptide (GSGS) that distances the GFP moiety from the rest of the BAG3, as reported in [75]; WT, wild type. (B, D) BAG3-GFP and GFP IPs were prepared from HeLa cells infected with recombinant adenoviruses driving expression of BAG3-GFP or GFP proteins (Ad-BAG3-GFP or Ad-GFP) using anti-GFP, and analyzed by Western blot; levels of endogenous BAG3, HSC70 and HSPB8 in total cell extracts are shown (Input). (C) Schematic of the protocol developed to achieve knockdown-rescue experiments using siBAG3_3, Ad-BAG3-GFP and BacMam-RFP-a-tubulin. Total cell extracts were prepared 46 h after transfection during the 2^nd^ release and analyzed by Western blot with anti-BAG3 and anti-HSPB8 antibodies. Please note that individual BAG3-GFP proteins were introduced at near the endogenous level of BAG3 and could restore HSPB8 levels in BAG3-depleted cells, except for BAG3 (IPV) unable to bind to HSPB8. (E) Representative confocal time-lapse sequences of RFP-a-tubulin in cells from C, showing restoration of spindle dynamics upon introduction of WT BAG3-GFP, but not of BAG3-GFP (IPV) in BAG3-depleted cells; red and yellow asterisks designate the position of spindle poles (S5 and S6 Movies); Bars, 10 μm. Mitotic cells were imaged by spinning disk confocal microscopy for 60 to 90 min at ~1.5 min intervals. Please refer to S4–S8 Movies for samples of the phenotypes observed. (F, G) Quantification of cells from C, indicating percentages of cells with abnormal mitosis defined as spindle rocking, stalled in mitosis +/- spindle rocking, or chromosome misalignment; means +/- SE from 88 to 184 cells from 3 to 5 independent experiments as indicated (S1 Dataset). Statistical significance was analyzed with the Exact Fisher Test; (F) siBAG3_3 + WT versus siBAG3_3 + GFP: ****, p < 0.0001; siBAG3_3 + WT versus siBAG3_3 + IPV: **, p = 0.001; siBAG3_3 + WT versus siBAG3_3 + ΔPXXP: ***, p = 0.0003; siBAG3_3 + WT versus siBAG3_3 + ΔBAG: ***, p = 0.003; (G) siBAG3_3 + WT versus siBAG3_3 + GFP: *, p = 0.005; siBAG3_3 + WT versus siBAG3_3 + R480A: ns, p = 0.884; siBAG3_3 + WT versus siCtl + GFP: ns, p = 0.46.

We next sought to determine the dependence of the mitotic function of BAG3 for its associated molecular chaperones and for its PXXP motif, which connects BAG3 to signaling factors [37]. To do so, we developed a protocol to perform depletion-rescue experiments. Structural requirements on BAG3 for restoration of normal spindle dynamics were investigated by using BAG3-GFP fusion proteins bearing mutations that abolish binding to HSPB8 (IPV) [38], deletion of the HSP70/HSC70-binding domain (ΔBAG) or deletion of the PXXP motif (ΔPXXP) (Fig 4A) [39, 40]. Recombinant adenoviruses were used to express the fusion proteins, which were appropriately localized within cells and exhibited the expected behaviors in terms of binding to HSPB8 and HSP70/HSC70 (Fig 4B and S2 Fig). Using siRNA duplex targeting the 3’ untranslated region of BAG3 and the experimental scheme depicted in Fig 4C, we achieved efficient knockdown of endogenous BAG3. Individual BAG3-GFP proteins were introduced at near physiological levels in BAG3-depleted cells (Fig 4C) together with RFP-a-tubulin to determine their functional impact on spindle dynamics in single cells. Under these conditions, expression of wild type (WT) BAG3-GFP in BAG3-depleted cells, but not of GFP, could restore to near normal the progression through early mitosis, bringing the level of phenotypic defects in line with control cells (~37% compared to ~32%; Fig 4E and 4F; S4 and S5 Movies). It also corrected the decrease seen in HSPB8 protein levels upon depletion of BAG3, consistent with our previous findings that BAG3 and HSPB8 form a stoichiometric complex that is destabilized upon reduction of BAG3 levels (Fig 4C, compare GFP to WT) [15]. In marked contrast, BAG3 (IPV) could not efficiently restore HSPB8 levels nor correct the mitotic defects in BAG3-depleted cells, despite binding to HSP70/HSC70 (Fig 4B). This was reflected by a persistence of characteristic mitotic defects (delays in metaphase with spindle rocking) in a significant proportion of BAG3-depleted cells expressing BAG3 (IPV) (Fig 4E and 4F; S6 Movie). Likewise, BAG3 (ΔPXXP) was unable to correct the BAG3-dependent mitotic phenotype (Fig 4F), despite binding to HSPB8 and restoring its protein levels (Fig 4B and 4C). This suggests that interactions with HSPB8 and other proteins through the PXXP motif are required to mediate the effect of BAG3 on mitotic spindle positioning.

Expression of BAG3 (ΔBAG) also could not restore normal spindle dynamics (Fig 4F). However, the BAG domain might regulate BAG3 interactions with proteins other than HSP70/HSC70 [39, 41, 42]. To more specifically ascertain the contribution of HSP70/HSC70, we introduced a point mutation in the BAG domain of BAG3 (R480A) that disrupts its association with HSP70/HSC70 but not its binding to SH3-containing proteins (Fig 4D, upper panel) [43]. Surprisingly, BAG3 (R480A) expression significantly reduced the characteristic mitotic defects in cells treated with BAG3-specific siRNAs (Fig 4D, lower panel, and Fig 4G and S7 Movie). In contrast, this mutant BAG3 (R480A) failed to restore aggresome-targeting of ubiquitinated proteins aggregates (S3 Fig). Thus the data suggest that the mitotic effects of BAG3 mostly rely on its association with HSPB8 and that binding to HSP70/HSC70 is dispensable.

**Fig 5:**
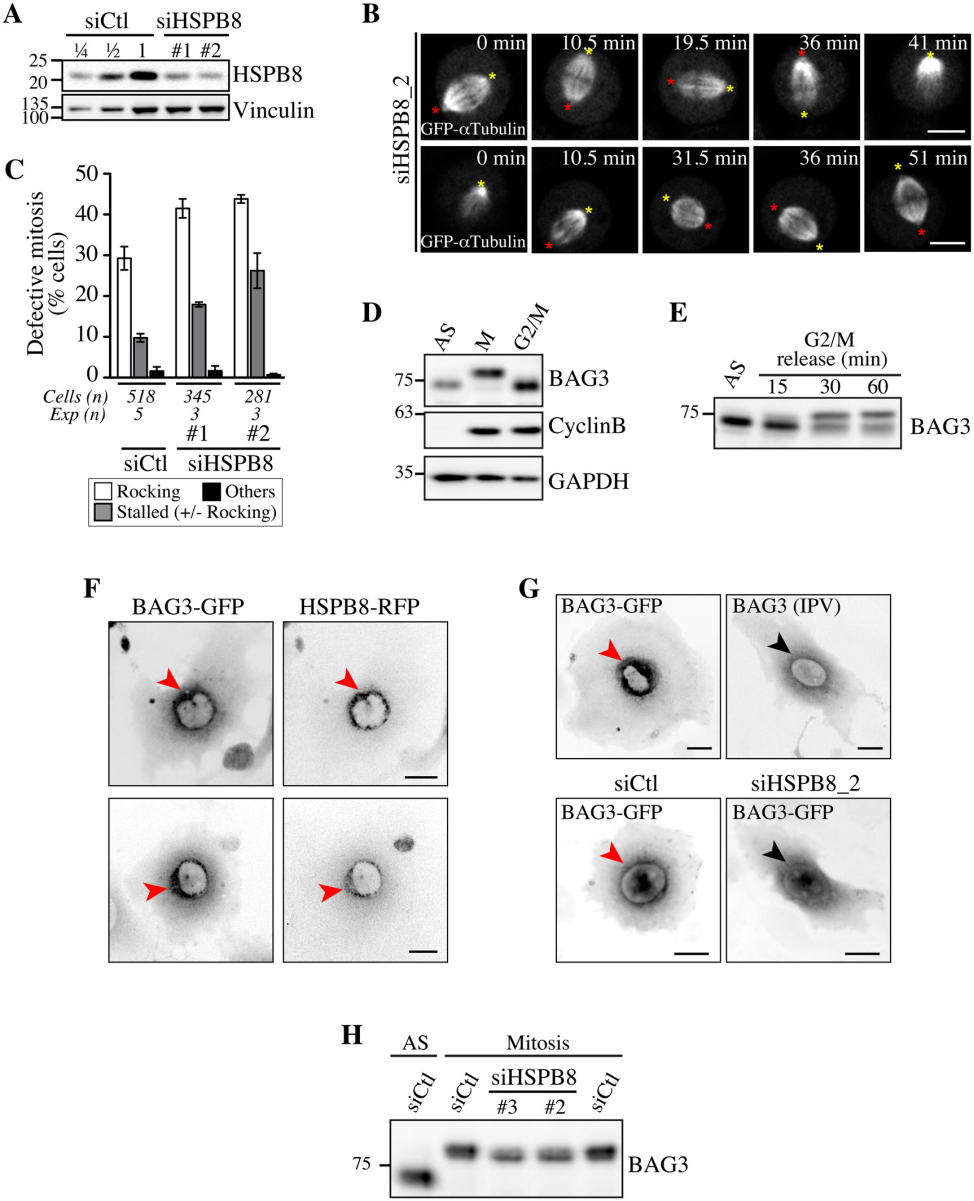
Defects in spindle dynamics upon depletion of HSPB8 correlate with impaired localization of BAG3 at G2/M. (A) Representative Western blots of total cell extracts prepared from HeLa-RFP-H2B transfected with control siRNA (siCtl) or HSPB8-specific siRNAs (siHSPB8 #1 and #2), showing reduction of HSPB8 levels of ~75%; vinculin levels: loading controls. (B) Confocal time-lapse sequences from HeLa-RFP-H2B cells transfected with siHSPB8_2 and transduced with BacMam-GFP-a-tubulin, which show aberrant spindle rocking; colored asterisks designate spindle poles. Cells were synchronized by the double Thymidine block method and imaged by spinning disk confocal microscopy for 60 min at ~1.5 min intervals; Bars, 10 μm. (C) Quantification of cells from B, indicating abnormal mitosis defined as spindle rocking, stalled in mitosis +/- spindle rocking, or others (chromosome misalignment or multipolar spindle); means +/- SE from at least 281 cells from at least 3 independent experiments, as indicated (S1 Dataset). (D-E) Western blots of extracts prepared from asynchronous HeLa-RFP-H2B cells (AS), arrested in mitosis by a nocodazole-block (100 ng/ml, 16 h), or arrested at the G2/M border by a Thymidine/RO-3306-block (8 μM RO-3306, 18 h), showing hypophosphorylated BAG3 (AS, G2/M), hyperphosphorylated BAG3 (M), and cyclin B1. (E) RO-3306-treated cells that have been released in fresh medium show BAG3 supershifted band as cells exited the G2-block; AS: asynchronous. GAPDH levels: loading controls. (F, G) Deconvolved image stacks of G2-arrested HeLa cells transducted with Ad-BAG3-GFP and Ad-HSPB8-RFP, showing robust enrichment of BAG3-HSPB8 to centrosomes and the surrounding perinuclear material (red arrows). Please note that mutation of HSPB-binding domains (BAG3 IPV) like depletion of HSPB8 (siHSPB8) markedly impaired recruitment of BAG3-GFP to a defined perinuclear space at G2/M (black arrows). (H) Western blots of total extracts prepared from siRNA-treated HeLa cells arrested at prometaphase by treatment with nocodazole (100 ng/ml, 16 h), showing partial supershift of BAG3 in cells depleted of HSPB8; AS: asynchronous cells.

To further confirm a role for HSPB8, analyses of spindle dynamics were performed after treating HeLa cells with HSPB8-specific siRNAs that achieved ~75% depletion of HSPB8 in these cells (Fig 5A). In agreement with a role as a mitotic partner of BAG3, depletion of HSPB8 delayed mitotic progression (S4A Fig). Time-lapse imaging showed dramatic spindle movements in the *xy* plane and *z*-axis rotations in cells treated with different HSPB8-specific siRNAs, together with defects in mitotic spindle positioning that mimicked the BAG3-dependent mitotic phenotype (Fig 5B and 5C; S9, S10 and S11 Movies). Remarkably, HSPB8-RFP, unlike RFP, showed robust recruitment to centrosomes and the surrounding perinuclear material in cells arrested at the G2/M border by treatment with the specific cyclin-dependent kinase 1 (CDK1) inhibitor RO-3306 (Fig 5F, red arrows, and S4C Fig) [44]. In agreement with BAG3 translocation to the centrosomal region before nuclear envelope breakdown during an unperturbed mitosis (Fig 1E), both HSPB8 and hypophosphorylated BAG3 were confined to a spherical perinuclear space in G2-arrested cells (Fig 5D and 5F). However, BAG3 (IPV) like wild type BAG3-GFP in cells depleted of HSPB8 remained more diffuse in the perinuclear space of G2-arrested cells (Fig 5G, black arrow). In addition, HSPB8 depletion reduced BAG3 supershift in mitotic cells (Fig 5H, siHSPB8, and S4D Fig, IPV). Thus HSPB8 appeared to facilitate BAG3 translocation to a centromal-perinuclear region before nuclear envelope breakdown and its subsequent phosphorylation at mitotic entry, suggesting that both events are related. Based on the results, we conclude that the effect of BAG3 on mitotic spindle orientation requires its interaction with HSPB8 and some signaling function of the PXXP motif, but not binding to HSP70/HSC70.

### Silencing of the autophagy cargo receptor p62/SQSTM1 mimics the effect of BAG3 and HSPB8 depletion on mitotic spindle dynamics

**Fig 6:**
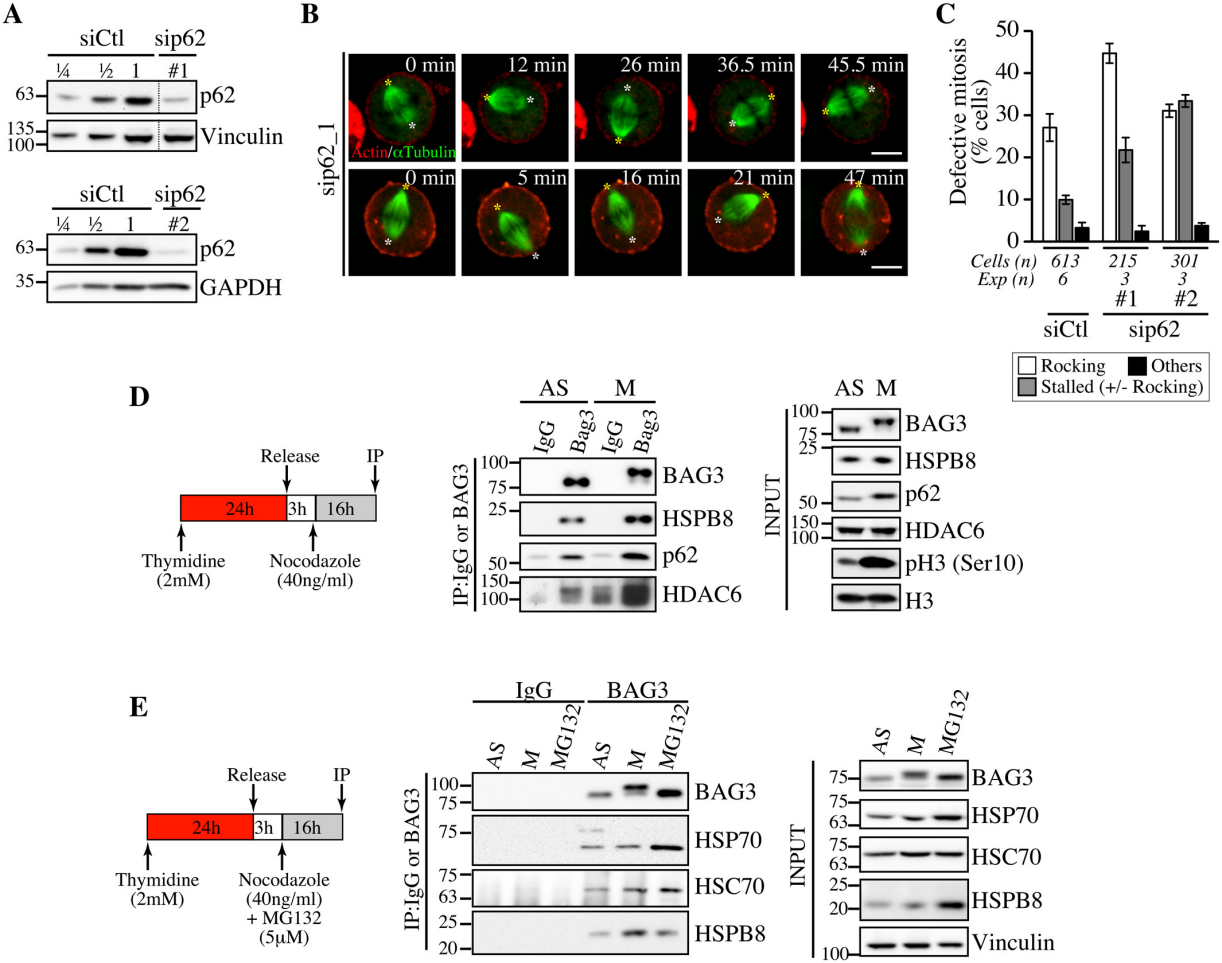
Depletion of p62, which forms a mitotic complex with BAG3-HSPB8, recapitulates the same phenotype on spindle dynamics. (A) Representative Western blots of total extracts prepared from HeLa-RFP-H2B cells transfected with control siRNA (siCtl) or p62-specific siRNAs (sip62 #1, #2); vinculin and GAPDH levels: loading controls. (B) Confocal time-lapse sequences from HeLa-RFP-H2B cells transfected with sip62_1 and transduced with BacMam-GFP-a-tubulin and BacMam-RFP-actin, which show aberrant spindle rocking; colored asterisks designate spindle poles. Cells were synchronized by a double Thymidine block and imaged by spinning disk confocal microscopy for 60 min at ~1.5 min intervals; Bars, 10 μm. (C) Quantification of cells from B, indicating abnormal mitosis defined as spindle rocking, stalled in mitosis +/- spindle rocking, or others (chromosome misalignment or multipolar spindle); means +/- SE from at least 215 cells from at least 3 independent experiments, as indicated. (D, E) BAG3 IPs were prepared from asynchronous (AS) or from mitotic HeLa-RFP-H2B cells synchronized by a Thymidine/nocodazole block and recovered by mitotic shake off (M), or from cells treated with proteasome inhibitor for 16 h (MG132, 5 μM), using anti-BAG3 antibody or rabbit IgG, and analyzed by Western blot with the indicated antibodies. The levels of BAG3, HSPB8, p62, HDAC6, HSP70 and HSC70 in total cell extracts are shown (Input); phospho-histone H3 (pH3) and histone H3 levels: mitotic markers. The data are representative of at least 3 independent experiments.

We then examined a potential connection between the mitotic function of BAG3 and BAG3-mediated selective autophagy pathway, which involves its association with p62/sequestosome-1 (SQSTM1) (hereafter named p62) [16, 23, 45]. P62 is considered to act as a receptor for ubiquitinated proteins to sequester misfolded protein aggregates in cytoplasmic inclusions and promote their autophagic clearance by interacting with the forming autophagosome [46–49]. Intriguingly, it has been found that p62 is specifically phosphorylated at mitotic entry and regulates anaphase onset [50]. Hence we assessed the impact of p62-specific siRNAs on mitotic spindle dynamics. Remarkably, depletion of p62 mimicked the phenotypic defects seen upon reducing BAG3 or HSPB8 levels, delaying cells at prometaphase-metaphase and causing dramatic spindle rocking (Fig 6A, 6B and 6C and S4B Fig; S12 and S13 Movies). While the mitotic delay observed here upon p62 depletion is consistent with previous work, this is the first report of an effect of p62 on spindle orientation.

To further investigate a functional connection between BAG3, HSPB8 and p62 in mitotic cells, we performed coimmunoprecipitation analyses in HeLa cells growing asynchronously (*AS*), or synchronized in mitosis with nocodazole (*M*). As expected, the hyperphosphorylated form of BAG3 was associated with HSPB8 in mitotic cell extracts (Fig 6D and 6E). Furthermore, its associations with p62 and histone deacetylase 6 (HDAC6), a p62 partner in the aggresome-autophagy pathways [51], were clearly enhanced (Fig 6D). No significant increase in HSP70/HSC70 was detected in mitotic BAG3 complexes, in agreement with an HSP70/HSC70-independent function for BAG3 in mitosis. In contrast, the BAG3 complexes isolated from cells submitted to proteasomal stress contained higher levels of HSP70 (Fig 6E). Together, these findings provide strong support for the existence of mitotic-specific BAG3 complexes comprising HSPB8 and p62 but not HSP70/HSC70 that would facilitate the accurate positioning of the mitotic spindle in HeLa cells.

### Knockdown of BAG3, HSPB8, or p62 impaired actin-based remodeling of mitotic cells and assembly of adhesion landmarks that regulate spindle orientation

Given that the BAG3-HSPB8 chaperone complex manages, together with p62, the remodeling of the actin-based cytoskeleton during mechanical stress [52], we hypothesized that their function in mitosis may similarly be related to the remodeling of actin-based structures. Spindle alignment depends on interactions between the spindle and the mitotic actin-based cytoskeleton, which undergoes extensive remodeling at mitotic entry to allow for changes in cell-shape. [53, 54]. These changes critically rely on the construction of a mechanically rigid actin cortex and the remodeling of mechanosensitive cell-substrate adhesions, for supporting the polarized distribution of force generator complexes to control interactions with astral microtubules [31, 55]. Because cells experience profound changes in cell tension homeostasis at mitotic entry, we reasoned that the chaperone complex could contribute to the “fitness” of actin-based structures that control spindle positioning.

**Fig 7:**
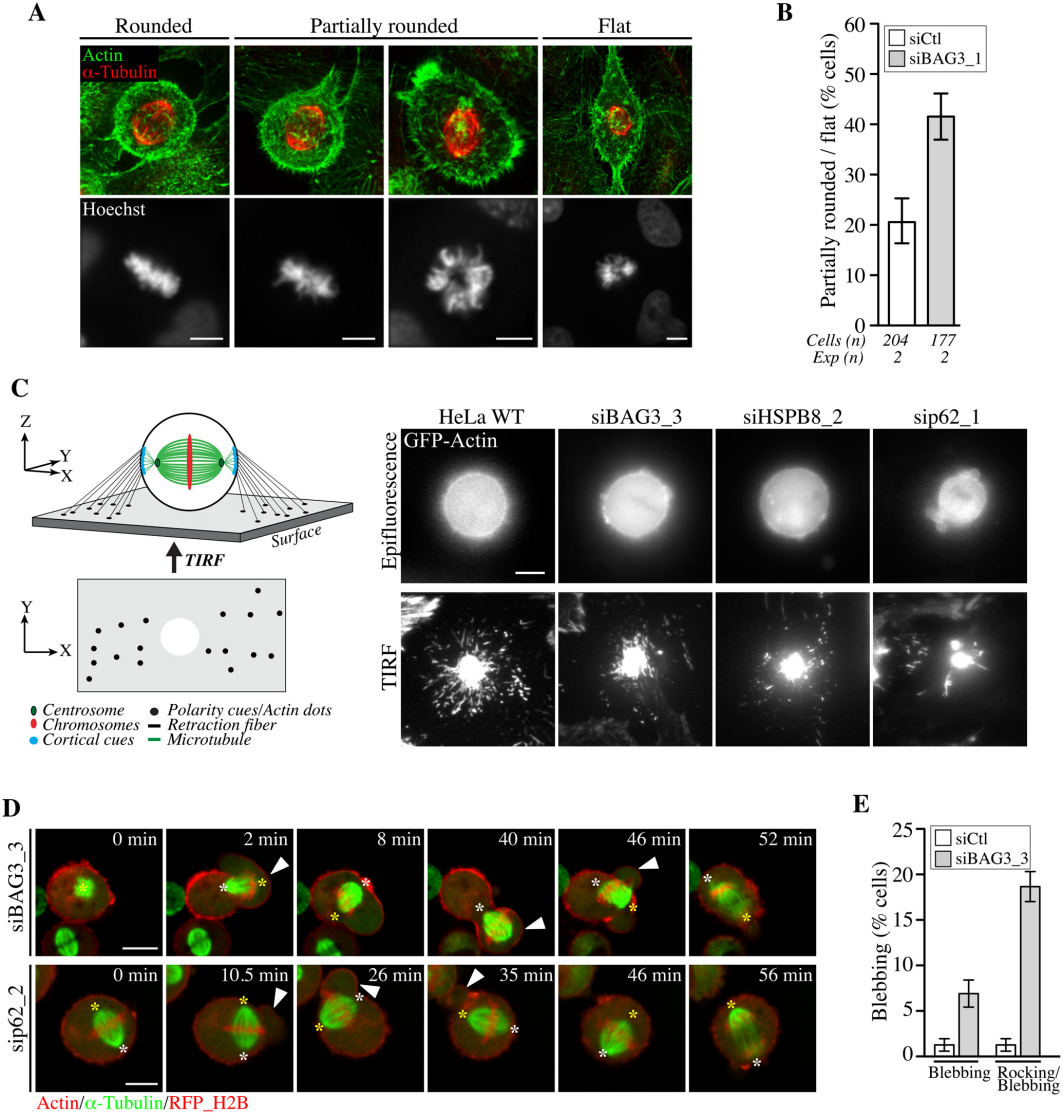
BAG3, HSPB8 and p62 regulate remodeling of mitotic F-actin structures. (A) HeLa cells transfected with control siRNA or with BAG3-specific siRNA (siBAG3_1) were synchronized by the double Thymidine block method and processed for IF with anti-a-tubulin, phalloidin (F-actin) and Hoechst (DNA). Deconvolved confocal image stacks show F-actin and chromosomes in rounded mitotic cells compared to partially rounded and flat mitotic cells; Bars, 10 μm (B) Quantification of cells from A, indicating the proportion of siRNA-treated cells with defects in mitotic cell rounding; means +/- SD from 2 independent experiments. (C) Schemes depicting the proposed organization of retraction fibers in *x*, *y*, *z* view, with the deduced *x*, *y* view by TIRFM of actin dots connecting the mitotic cortex to the adhesive substrate. Representative TIRFM images from HeLa cells at metaphase transfected with the indicated siRNAs and transduced with BacMam-GFP-actin, showing a bright actin cortex sitting on the adhesive substrate surrounded by numerous actin dots in control cells, which are disorganized upon depletion of BAG3, HSPB8 or p62; the corresponding epifluorescence images are shown. Cells were imaged 48 h after transfection of siRNAs. (D) Confocal time-lapse sequences of Hela-RFP-H2B cells at metaphase that have been transfected with siBAG3_3 or sip62_1 and transduced with BacMam-GFP-a-tubulin and BacMam-RFP-actin, showing abnormal blebbing of the mitotic cortex designated by arrowheads which is associated with displacements of the spindle (S14, S15 and S16 Movies); asterisks designate spindle poles; Bars, 10 μm. (E) Quantification of cells from D, indicating the proportion of cells undergoing blebbing alone or blebbing with spindle rocking; means +/- SE from 158 (siCtl) or 195 cells (siBAG3) from 3 independent experiments (S1 Dataset).

To test this idea, we first inspected the morphology of siRNA-treated HeLa cells synchronized in early mitosis. At onset of mitosis, cells retract and round up upon extensive remodeling of the actin-based cytoskeleton, leaving thin actin cables attached to the substrate called retraction fibers. The actin-rich retraction fibers are believed to act as guides to orient the spindle parallel to the adhesive substrate in tissue culture cells, by enabling cells to read changes in the mechanics of matrix to which they are attached and by providing polarity cues [34, 56]. Analysis of fixed cells stained with phalloidin (F-actin), anti-a-tubulin (spindle microtubules) and Hoechst (chromosomes) revealed a ~2-fold increase of flat or only partially round metaphase cells upon depletion of BAG3 (Fig 7A and 7B). Consistently, such cells often exhibited tilted mitotic spindles or unaligned chromosomes. This supported a role for BAG3 in timely remodeling of the actin-based cytoskeleton that directs cell rounding at mitotic entry [57, 58].

We then sought to analyze potential effect on the formation of actin-rich retraction fibers, which were proposed to convey large traction forces for spindle orientation [56]. We took advantage of total internal reflection fluorescence microscopy (TIRFM) to visualize how retraction fibers connect the mitotic cortex to the adhesive surface in live cells expressing GFP-actin (Fig 7C). Control cells at metaphase exhibited numerous actin dots that were distributed around the ventral cell cortex marked by a dense core of actin. These actin dots were presumed to represent the extremities of retraction fibers linking the mitotic cortex to the adhesive substrate, as they are described as membrane tubes filled with actin filaments (Fig 7C, scheme; HeLa WT) [59]. When BAG3, HSPB8, or p62 were silenced, there was a marked disorganization of the actin dots surrounding the ventral cortex of round, mitotic cells (Fig 7C, siBAG3; siHSPB8; sip62). The actin dots now appeared reduced in numbers and/or dispersed in small clusters instead of showing a circumferential distribution. Examination of retraction fibers by confocal microscopy in mitotic cells expressing LifeAct-GFP—a probe that binds to F-actin, confirmed a marked decrease in retraction fibers surrounding the mitotic cortex of BAG3-depleted cells (S5D Fig). In agreement with a specific role for BAG3, depletion of BAG1 or BAG2—two other BAG proteins devoid of HSPB8-binding motif [15], did not similarly affect retraction fiber distribution (S5A, S5C and S5D Fig). Moreover, unlike BAG3 neither BAG1 nor BAG2 showed detectable supershifted band in mitotic cell extracts (S5B Fig). Thus the data strongly suggest that mitotic rounding, the process by which retraction fibers are formed, is specifically impaired upon reduction of BAG3, HSPB8 or p62 levels.

We further observed that depletion of BAG3 or p62 enhanced membrane blebbing and cell-shape deformations in prometaphase-metaphase cells, arguing for deregulation of cortical tension even in the absence of spindle phenotype (Fig 7D and 7E). Remarkably, in cells showing severe phenotypes, spindles were seen erratically rocking or pushed toward blebs of unusually large size (Fig 7D, time-sequences; S14, S15 and S16 Movies). Such phenotypes were reminiscent of mitotic defects caused by disrupting cortical cues/anchors directing stable spindle positioning.

### Stiffening of the mitotic cortex can correct spindle dynamics defects caused by depletion of BAG3

**Fig 8:**
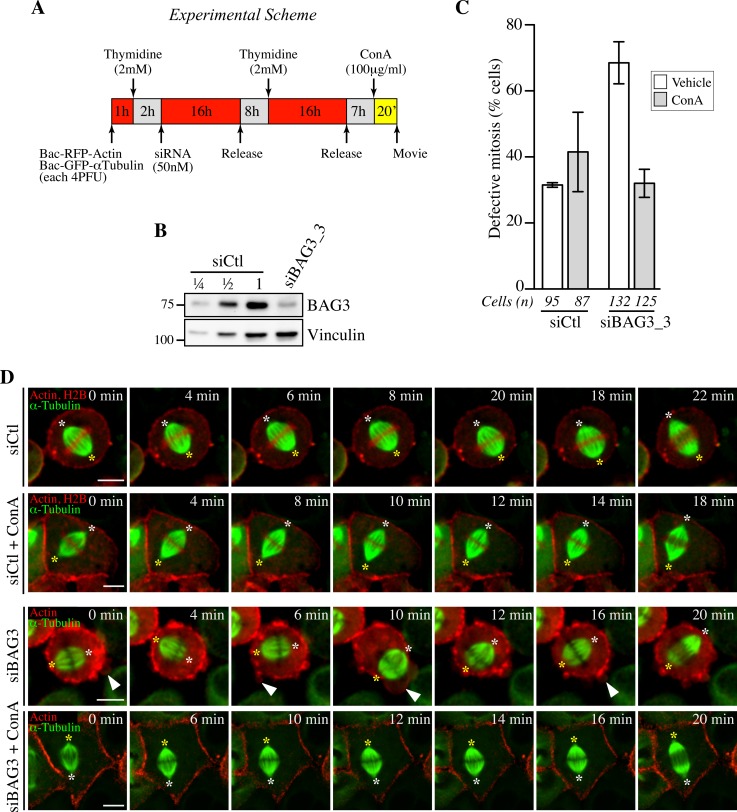
Concanavilin A restores normal spindle dynamics in BAG3-depleted cells. (A) Experimental scheme. (B) Western blot of total cells extracts prepared from HeLa-RFP-H2B cells transfected with control siRNA (siCtl) or siBAG3_3 and processed as depicted in A. (C) Quantification of the proportion of siRNA-treated cells that exhibited abnormal mitosis after a 20-min incubation period with ConA (100 μg/ml) or the vehicle, defined as spindle rocking, mitotic arrest +/- rocking, cortex blebbing and/or chromosome misalignment; means +/- SD from at least 87 cells from 2 independent experiments (S1 Dataset). Cells were imaged by spinning confocal microscopy for 60 min at ~ 2 min intervals. (D) Representative confocal time-lapse sequences from HeLa-RFP-H2B cells from C, showing that characteristic spindle rocking associated cortical blebbing is inhibited upon addition of ConA; please note that concanavilin A impaired mitotic cell rounding in both control HeLa-RFP-H2B cells transfected with siCtl and in siBAG3-treated cells, as reported before [58]. Arrowheads designate cortex blebbing in siBAG3-treated cells incubated with the vehicle only; asterisks designate spindle poles; Bar, 10 μm.

Cortical stiffening is associated with cell rounding at mitotic entry and is thought to help stabilize cortical cues and the mitotic spindle. Hence the phenotypes observed upon depletion of BAG3, HSPB8 or p62 could be due, at least in part, to defects in establishing a mechanically rigid actin cortex. To obtain evidence for causal relationship, we incubated siRNA-treated cells synchronized in mitosis in medium containing the tetravalent lectin concanavalin A for a short period of time (Fig 8A and 8B). Concanavalin A extensively crosslinks glycoproteins from the plasma membrane and is often used to increase stiffness of the cortex from the outside [58, 60, 61]. Significantly, this treatment stabilized spindle positioning in BAG3-depleted cells and led to a 2.5-fold reduction in the number of cells at metaphase showing defects in spindle positioning upon depletion of BAG3, bringing it in line with cells transfected with control siRNAs (Fig 8C and 8D). Collectively, our results suggest that defects in spindle orientation are functionally related to an inability to establish a rigid mitotic cell cortex and to generate the appropriate tensile forces.

## Discussion

BAG3 and HSPB8 form a stoichiometric complex that has been linked to protein quality control. In this study, we present evidence for a novel role for the BAG3-HSPB8 complex in remodeling of the actin-based cytoskeleton at mitotic entry, which involves possible interactions with signaling proteins through BAG3 PXXP motif and is mimicked by the autophagy receptor p62. The exact mechanism whereby BAG3-HSPB8 can regulate mitotic cell remodeling remains to be defined at the molecular level. Because the same mitotic phenotype is recapitulated by depleting any protein in the BAG3-HSPB8-p62 axis, we suggest that they act within a so-far unrecognized chaperone-mediated quality control mechanism to assist in the proper and timely assembly of a rigid actin cortex that is proposed to maintain high mechanical stress throughout metaphase to guide spindle orientation. To our knowledge, this is the first report of a role for BAG3-HSPB8 in the normal operation of dividing cells, one for which HSP70/HSC70 would be dispensable.

Our finding that BAG3-HSPB8 regulates mitotic cell rounding and the integrity of mechanically sensitive actin-rich retraction fibers is in line with the reported role of BAG3-HSPB8 as a component of a multichaperone complex that regulates a selective form of autophagy called chaperone-assisted selective autophagy (CASA). CASA has been described as a tension-induced autophagy pathway for removal and degradation of large cytoskeleton components damaged during contraction in muscle cells to promote stability of F-actin-based structures [62]. In this paradigm, HSPB8 is proposed to be required for efficient recognition of the actin-crosslinking protein filamin C, which becomes mechanically unfolded [52]. HSC70 would mediate processing of substrate proteins by recruiting an E3 ubiquitin ligase, providing a signal for the autophagic receptor p62. Even though mitosis and muscle contraction are completely unrelated cellular processes, they share a requirement for maintaining the integrity of actin-based structures under high tension. It has been shown by Fink et al. that the mitotic actin cortex is a unique structure that combines mechanosensivity with robustness to protect the process of spindle formation from external perturbations [56]. The results presented here suggest that changes in tension homeostasis at mitotic entry could engage a specialized BAG3-HSPB8 chaperone activity to assist in the construction of a mechanically robust actin cortex and of functional retraction fibers, which are crucial to orient centrosomes and mediate proper spindle positioning. Recruitment of the BAG3-HSPB8 complex to centrosomes and the surrounding perinuclear material at the G2/M border is consistent with such a role.

Despite some similarities with the CASA process, we demonstrate that the activity of BAG3-HSPB8 in early mitosis is uncoupled from the HSP70/HSC70 chaperone system, suggesting a different mode of action and reinforcing the notion that not all HSPBs cellular activities would require a functional HSP70/HSC70 chaperone machine [11, 63]. Indeed, the mutant BAG3 (R480A) that cannot bind to HSP70/HSC70 was able to rescue the mitotic activity of BAG3 on spindle dynamics; this mutant was however defective in the aggresome-autophagy pathway, as expected. In contrast, a mutant BAG3 bearing large deletion of the BAG domain was defective in our model, which might reflect additional partners for the BAG domain as suggested before [39]. BAG3 is specifically hyperphosphorylated at mitotic entry, just like p62 [50], suggesting that phosphorylation could favor mitotic-specific protein interactions. In agreement, associations between BAG3, HSPB8, p62 and HDAC6 were promoted in mitotic cells, while BAG3 association with HSP70/HSC70 was more abundant under conditions of proteasomal failure. Thus a specialized chaperone complex comprising BAG3-HSPB8-p62 (but not HSP70) may be activated by cell cycle-regulated kinases to facilitate the detection and sequestration of cytoskeletal/signaling components that need to be removed for timely remodeling of mitotic actin-based structures. While the ATP-independent holdase function of the HSPB family is thought to sequester damaged proteins to prevent further aggregation during proteotoxic stress, it appears also to be exploited under normal conditions as a reversible mechanism to sequester proteins [8, 9]. For instance, HSPB1 would sequester actin monomers under resting conditions and serve as a “reservoir” of actin, which would be released upon agonist-induced phosphorylation of HSPB1 to promote local actin assembly [64]. Recent findings support a role for the ubiquitin system in the targeting and removal of a cytoskeletal adaptor protein from the cortex at the G2/M border to favor mitotic changes in actin network architecture [65]. This suggests the existence of cell cycle-regulated mechanisms for recognition of cytoskeletal proteins that need to be segregated at a precise time. BAG3, HSPB8 and p62 are good candidate components of such mechanism. Of note, autophagy, like almost all membrane transport processes, is downregulated at mitotic entry and resumes to some extent during cytokinesis [66, 67]. Therefore, segregation of cytoskeletal components to inclusions could serve to remove proteins from their functionally relevant site(s) in early mitosis, at a time when the burden on the ubiquitin-proteasome system is high, until they can be properly disposed or recycled after mitotic exit.

While the link with p62 suggests a mechanism related to the activity of BAG3-HSPB8 in autophagic sequestration, it might also be that the BAG3-HSPB8 complex participates to mitotic cell remodeling via independent, non-canonical mechanism. The mitotic effect of BAG3 was found here to depend on BAG3 PXXP motif, which mediates interactions with the Src homology 3 (SH3) domain of a number of signaling proteins that are components of actin-based motility pathways. For instance, BAG3, via its PXXP motif, interacts with the SH3 domain of the oncogenic tyrosine kinase Src, a crucial signaling hub controlling cell shape that promotes spindle orientation in early prometaphase [37, 68, 69]. Thus an HSPB8-dependent activity of BAG3 could provide a platform to facilitate recruitment and assembly-disassembly of mitotic-specific cytoskeletal complexes, similar to the HSC70-dependent effect of BAG3 in muscle cells, which promotes recruitment of CapZ to the Z-disk and stability of F-actin [22]. HSPB8 was found here to facilitate proper localization of BAG3 at the G2/M phase and its subsequent phosphorylation at mitotic entry, perhaps on residues that could alter PXXP-dependent interactions. A systematic analysis of the contribution of site-specific phosphorylation events on BAG3 is warranted. The autophagic sequestration and the scaffolding models are not mutually exclusive and could contribute for the mitotic effects of BAG3-HSPB8. Beyond its role in protein storage or turnover, p62 acts also as a protein scaffold for the formation of multiprotein complexes in various signaling pathways [70, 71]. Regardless of the exact mode of action of BAG3-HSPB8-p62 axis in mitotic cellular remodeling, we can reasonably speculate that BAG3 PXXP motif serves to recruit BAG3-HSPB8 chaperone complex to machineries controlling remodeling of the cytoskeleton at mitotic entry.

The existence of a tertiary complex formed by BAG3-HSPB8-p62 in mitotic cells is strongly supported by the data presented here. Depletion of either, BAG3, p62 or HSBP8, results in the same phenotype in mitotic cells. The effect seen at the level of retraction fibers is uncommon and highly unlikely to be nonspecific. Indeed, depletion of other BAG proteins did not generate the same mitotic defects at the level of retraction fibers. This is further bolstered by interaction data showing that BAG3 associations with HSPB8 and p62, but not with HSP70, are promoted in mitotic cell extracts. The molecular mode of interaction between BAG3 and p62 is unknown, but may include a number of shared protein partners. Notably, recruitment of HDAC6, another p62 partner protein, was also significantly enhanced in mitotic BAG3 complexes. To our knowledge, this is the first report of a link between BAG3 and HDAC6, a unique histone deacetylase that binds to dynein [72]. Beyond its role in the segregation and aggresome-targeting of misfolded proteins, HDAC6 possesses several cytoskeletal substrates of relevance for remodeling of actin-based mitotic structures and spindle orientation, including the actin-binding protein cortactin and a-tubulin [73, 74]. A potential interaction between BAG3 and dynein, which has been reported before, could be meaningful for spindle orientation [16, 53]. While we cannot exclude some effects on microtubule dynamics upon depletion of BAG3, we did not observe major changes in the levels and organization of spindle and astral microtubules (S6 Fig). Accordingly, we propose that actin-based structures are the prime targets of this novel mitotic activity regulated by BAG3, based on the severe phenotypic changes observed at the level of actin-rich retraction fibers, cell morphology and cortex stability (e.g. blebbing) upon depletion of BAG3, HSPB8 or p62.

In conclusion, we have uncovered a novel role for BAG3-HSPB8 chaperone complex in mitotic cell-shape changes, spindle orientation and proper chromosome segregation in the context of rapidly dividing cells. Such function could be meaningful for tumor cell survival and their uncontrolled capacity to divide. Conceptually, engagement of a specialized chaperone activity in rapidly dividing tumor cells at mitosis could serve several purposes. It could prevent aggregation of cytoskeletal proteins damaged by repetitive cycles of polymerization-depolymerization, assist in the assembly of macromolecular complexes responsible for actin-based dynamics and/or promote the sequestration and/or autophagic elimination of damaged components of mitotic actin structures that are proposed to be under high stress throughout metaphase. Elucidation of the precise mechanism involved should contribute to further our understanding of the apparent addiction of some cancer cells to the co-chaperone activity of BAG3 that might support uncontrolled cell division in the face of high mitotic stress experienced by most malignant cells.

## Materials and Methods

### Expression vectors, recombinant adenoviruses and baculoviruses

BAG3-GFP constructs were obtained by subcloning the EcoRI/BamHI fragments of myc-BAG3 constructs (WT, BAG3 [Δ421–498] or IPV [I96V/V98G/I208G/V210G] (described in [15, 38]) into the pEGFP-N1 vector (Clontech laboratories Inc.). To circumvent possible folding problems upon direct fusion of GFP to BAG3, GFP sequences were fused to the carboxyl terminus of BAG3 by engineering a glycine/serine-rich flexible linker (GSGS) peptide that distanced the GFP moiety from the rest of the BAG3 protein as described [75]. BAG3 ΔPXXP (Δ302–418) and BAG3 (R480A) were generated by PCR using the resulting BAG3-GFP as template and the following primers: 302-rev: 5’-CCT GTC GAC CAC GGT GTG CAC ACG G-3’ and 418-gen: 5’-Phosphate-CAT CCA GGA GTG CTG AAA GTG GAA GCC-3’; 480A-gen: 5’-GAT GTG CGT CAG GCC GCG AGA GAC GGT GTC-AG-3’ and 5’-CTG ACA CCG TCT CTT GCG GCC TGA CGC ACA TC-3’. HSPB8-RFP bears a glycine/serine-rich flexible linker peptide (GSGS) that distances the RFP moiety from the rest of the HSPB8 protein and was generated by subcloning the BglII/KpNI fragment of human HSPB8 that had been amplified in phase with a GSGS linker by PCR, in pmRFP-C1 (Clontech laboratories Inc.). The following primers were used: HSPB8.gen 5’-GAA GAT CTG AAA TGG CTG ACG GTC AGA TGC CCT TCT CC-3’ HSPB8gsgs.rev 5’-CGG GTA CCG CCG AGC CGG TAC AGG TGA CTT CCT GGC TGT CCT G-3’. All constructs were confirmed by DNA sequence analyses. Recombinant adenoviruses for expression of BAG3-GFP proteins and HSPB8-RFP as single gene products were produced by Welgen Inc, Worcester, MA, and were amplified in the HEK293VR, as described before [76]. All recombinant adenoviruses were confirmed by sequencing of the inserted sequences. Virus titers were determined using the AdenoX Rapid titer kit (Clontech Laboratories, #631028) following the manufacturer’s instructions. The CellLight BacMam 2.0 ready to use reagents Bac-GFP-actin (C10582), Bac-RFP-actin (C10583), Bac-RFP-αtubulin (C10614) and Bac-GFP-a-tubulin (C10509) (Life Technologies), and the Adenovirus LifeAct-TagGFP2 (60121, IBIDI), were utilized for live-cell imaging experiments.

### Antibodies and chemicals

The following antibodies and drugs were used: rabbit anti-BAG3 LP9 was raised against a C-terminal peptide (SSMTDTPGNPAAP), rabbit anti-BAG3 LP11 was raised against full length recombinant human BAG3 fused with glutathione S transferase (LP11), and rabbit anti-HSPB8 against a C-terminal peptide (NELPQDSQEVTCT) [15]; human anti-CREST (Immuno Vision); anti-HSC-70 (sc-1059), anti-HDAC6 (sc-11420), anti-cyclin B1 (sc-245), anti-GFP for Western blot (sc-9996), anti-Mad2 (sc-65492) and anti-p62 (sc-28359), anti-BAG1 (sc-33704) and anti-BAG2 (sc-366091) were from Santa Cruz Biotechnology; anti-α-tubulin (Abcam, ab18251); anti-a-tubulin (T5168), Hoechst Bisbenzimide H 33342 (B2261), anti-g-tubulin (T6557, Clone GTU-88) and anti-vinculin (V9131) were from Sigma; Alexa-Fluor 488 Phalloidin (A12379), Texas Red-X Phalloidin (T-7471) and anti-GFP for IP (A-11120) were from Molecular Probes/Thermo Fisher; anti-calreticulin (BD, #612136); anti-GAPDH Clone 6C5 (Fitzgerald, #10R-G109a); anti-phospho-Histone 3 Ser10 and anti-ubiquitin (clone FK2, #02–263) were from Millipore; anti-CEP170 (Invitrogen, #413200); anti-HSPB5/aB-crystallin (SPA222) and anti-HSP70 (SPA810) were from StressGene; anti-H3 (gift from A. Ruiz-Carrillo). Culture dishes were coated with Fibronectine (10 μg/ml; Sigma, F1141) or Poly-L-Lysine (1 mg/ml; Sigma, P1399). Sodium arsenite, E64d, pepstatin A, Cytochalasin D, RO-3306 (SML0569) and MG132: Z-leu-leu-leu-al (C2211) were from Sigma, and Rapamycin was from Calbiochem.

### Cell culture, transfection and cell synchronization

293T cells [77] were grown in Dulbecco’s modified Eagle’s medium (DMEM), supplemented with 10% Fetal Bovine Serum (FBS). HEK293VR cells contain the tetracycline repressor to allow efficient replication of recombinant adenoviruses carrying toxic genes (a generous gift from Philip E. Branton, McGill University) and were cultured in a-minimal essential medium (a-MEM) with 10% FBS. HeLa [78], HeLa-GFP-H2B (a generous gift from Laurence Pelletier) [79], and HeLa-RFP-H2B [80] cell lines were maintained in a-MEM with 10% FBS. MCF7 cells were maintained in a-MEM supplemented with 10% FBS. Cells were grown in a humidified atmosphere with 5% CO_2_ at 37°C. HeLa cells were transfected with Lipofectamine 2000 (Invitrogen) following the manufacturer’s instructions. HeLa cell lines were synchronized in G1 by a double Thymidine block and were released for 7 h in fresh medium. Briefly, cells were incubated with 2 mM Thymidine (Sigma, T9250) in a-MEM without Desoxyribonucleosides/Ribonucleosides supplemented with 10% FBS (aMEM minus) for 16 h, were washed 3-times and released in fresh medium. After the second block, cells were released in a-MEM supplemented with 10% FBS. Thymidine/nocodazole block was performed to increase the proportion of cells in mitosis by a 24 h-incubation period with 2 mM Thymidine followed by a 3 h-release period in fresh medium; cells were then synchronized in early mitosis by a 16 h-treatment with nocodazole (40 ng/ml; Calbiochem). Mitotic cell extracts were prepared from cells recovered mechanically by mitotic shake off. Cells were synchronized in G2/M by a 24 h-Thymidine block followed by a 3-h release in fresh medium and incubation with RO-3306 (8 μM) for 18 h.

### Knockdown-rescue experiments and siRNAs

For knockdown experiments, HeLa cells were transfected with 50 nM or 75 nM siRNA duplexes overnight using the calcium-phosphate method, MCF7 cells were transfected with 75 nM siRNA duplexes using Transit-TKO (Invitrogen) following the manufacturer’s instructions, and analyses were performed 48 h to 96 h later by Western blot or immunofluorescence. We developed a protocol called adenofection to perform depletion-rescue experiments and co-introduce low, non-interfering levels of GFP-actin and RFP-α-tubulin compatible with live cell imaging. HeLa-RFP-H2B cells seeded on fibronectin-coated glass dishes (MatTek Corp.) were infected with Ad-BAG3-GFP at a multiplicity of infection (MOI) of 2 plaque-forming units per cell (pfu/cell) together with BacMam 2.0 reagents at 4 pfu/cell in a-MEM minus medium containing 2 mM Thymidine. Two hours later, siRNA duplexes directed to the 3’-UTR (siBag3_3) were added in calcium-phosphate transfection buffer (50–75 nM in 125 mM MgCl_2_, 140 mM NaCl, 25 mM HEPES, 0.75 mM Na_2_HPO_4_). Simultaneous transfection-infection allows efficient virus transduction at low MOI. Sixteen hours post adenofection, cells were washed 3-times with HEPES (6.7 mM KCl, 150 mM NaCl, 10 mM HEPES, pH 7.3) and were released from the Thymidine block in fresh a-MEM minus medium for 7 h before adding back 2 mM Thymidine for a second 16 h-period. Cells were washed 3-times with phosphate buffer saline (PBS) and were released in complete a-MEM w/o Phenol Red. Live cell imaging was performed 7–9 h later. Where indicated in the figure legends, ConA (100 μg/ml; Sigma, L3885) was added 20 min before imaging. The siRNA duplexes were based on human sequences and were purchased from Qiagen (HPP grade siRNA) or Thermo Fisher Scientific (standard A4 grade). Sequences of the sense strands are as follows: siBag3_1: 5’-CGAAGAGTATTTGACCAAA-3’ (Qiagen); siBag3_2: 5’-GCAAAGAGGTGGATTCTAA-3’ (Thermo Fisher Scientific); siBag3_3: 5’-GATGTGTGCTTTAGGGAAT-3’ (Thermo Fisher Scientific); sip62_1: 5’-GGAAATGGGTCCACCAGGATT-3’ (Thermo Fisher Scientific); sip62_2: 5’-AGACCAAGAACTATGACAT-3’ (Thermo Fisher Scientific); siHspB8_1: 5’- CAGATAGGCTAGTGGTATT-3’ (Thermo Fisher Scientific); siHspB8_2: 5’-GCAGTGAATGCAAGGGTTATT-3’ (Thermo Fisher Scientific); siHspB8_3: 5’-CAGAGGAGTTGATGGTGAA-3’ (Thermo Fisher Scientific); Control siRNA (siCtl): AllStars Negative Control was purchased from Qiagen, target sequence: 5’-CAGGGTATCGACGATTACAAA-3’; siBAG1_1: 5’-AACCAGTTGTCCAAGACCT-3’; siBAG1_2: 5’-GCACGACCTTCATGTTACC-3’; siBAG2_2: 5’-AGCCAGGACATGAGGCAGA-3; siBAG2_3: 5’-TCAGAAGTTTCAATCCATA-3’.

### Cell fractionation, immunoprecipitation and phosphatase assay

Centrosomes isolation from asynchronous HeLa cells, or from cells arrested in mitosis by a 16 h-treatment with nocodazole (400 ng/ml), was performed essentially as described [81]. Briefly, cells extracts were prepared by hypotonic lysis et fractionated by sequential centrifugation on 50% sucrose cushion, followed by centrifugation on a step gradient of 70%, 50%, and 40% sucrose. The fractions containing centrosomes were centrifuged and centrosome pellets were processed for SDS-PAGE and Western blot. For immunoprecipitation, equal amounts of cells were washed in ice-cold PBS and were lysed by 3-cycles of freezing/thawing in 4-volume of lysis buffer (20 mM TRIS-HCl pH 7.6, 150 mM NaCl, 1 mM EDTA, 0,1% IGEPAL, 1 mM NaVO_4_, 10 mM NaF, 40 mM β-glycerophosphate, 1X Complete [Roche],, 1 mM DTT) (Figs 4D and 5G), or in NP40 buffer (20 mM TRIS-HCl pH 7.6, 150 mM NaCl, 1 mM EDTA, 1% IGEPAL, 1X Complete [Roche], 1 mM DTT) (Figs 1C and 4B). Cell extracts were centrifuged at 15,000 g for 15 min, the supernatants were transferred to a fresh tube and incubated with specific antibody for 60 min at 4°C, after which they were transferred to a tube containing the Dynabeads protein A (Life Technologies) pre-equilibrated in lysis buffer. After incubation for 30 min with gentle agitation, immuno-complexes were collected using a magnetic stand and washed 3-times in lysis buffer. Equal amounts of immune complexes were loaded on SDS-PAGE and analysed by Western blot as described before [82]. Phosphatase assay were performed in vitro by incubating BAG3 IPs in buffer (25 mM HEPES pH 7.5, 1 mM MnCl_2_, 5 mM NaCl, 0.005% Brij35, 1 mM DTT) supplemented with 200 unit of l-phosphatase (New England Biolabs) with or without phosphatase inhibitors (50 mM NaF, 10 mM NaVO_4_, 40 mM β-glycerophosphate, 50 mM EDTA), for 60 min at 30°C. BAG3 migration was analyzed on 8% SDS-PAGE, by Western blot. Protein concentrations were determined with DC protein assay reagent and densitometric analyses were performed from FluorS MAX MultiImager-captured images using the QuantityOne software version 4.5.0 (Bio-Rad Laboratories).

### Immunofluorescence, microscopy, live cell imaging and statistical analyses

DNA was stained with cell-permeable Hoechst and F-actin was stained with Alexa Fluor phalloidin (Molecular Probes/ Thermo Fisher Scientific, Ottawa, On. CA). For IF, cells were gently washed in Luftig buffer (0.2M sucrose, 35mM PIPES, pH 7.4, 5mM EGTA, 5mM MgSO_4_) and fixed in buffer containing 4% formaldehyde in 0.2% Triton X-100, 20 mM Pipes, pH 6.8, 1 mM MgCl_2_, 10 mM EGTA, pH 8.0, 4% formaldehyde for 10 min at RT. Specimens were then processed for IF as described [83], using the indicated primary antibodies and Alexa Fluor goat anti-rabbit or goat anti-mouse antibodies. Phenotypes were monitored routinely by at least two independent investigators by visual inspection of fixed specimens. Epifluorescence images were acquired with an AxioObserver Z1 system using a 40x Plan-Neofluoar 0.6NA objective and a charged-coupled device (CCD) camera Axiocam MRm controlled by the Zen software (Carl Zeiss). For measuring the *a* angle, centrosomes were stained with anti g-tubulin antibody. Confocal *z*-stacks were acquired at 0.5 μm steps and the spindle angle *a* was estimated from the spindle axis between the two centrosomes relative to the surface of the substrate according to the formula: *a* = 180°/π * arctan a/b. Confocal microscopy of live and fixed cells was performed with a Perkin Elmer UltraVIEW Spinning Disc Confocal (60x oil 1.4 NA, or 40x 0.75NA), equipped with an EMCCD cooled charge-coupled camera at -50°C (Hamamatsu Photonics K.K) and driven by Volocity software version 6.01. The system was equipped with a humidified/5% CO_2_/thermoregulated chamber. Mitotic phenotypes were estimated by visual inspection of time-sequences. For live cell imaging, HeLa-GFP-H2B or HeLa-RFP-H2B were seeded on fibronectin-coated glass dishes (MatTek Corporation, USA) and processed as indicated in the Figure legends before imaging using spinning disc confocal microscopy with a 40x 0.75 NA objective. Long-term imaging of asynchronous HeLa-GFP-H2B cells was performed using a Nikon TE-2000 inverted microscope equipped with a CO_2_/thermo-regulated chamber and a 40x 0.6 NA objective. Images were captured as 16 bit TIFF files with a Photometrix Coolsnap FX cooled CCD camera (-30°C) (Rooper Scientific, Tucson AZ) driven by the Metaview software version 4.5 (Universal Imaging Corp., Downingtown, PA). Images were acquired at 10 min intervals for a 48 h-period starting at 40 h posttransfection. Mitotic pedigrees for families of daughter cells were established by tracking the genealogy of individual cells, by visual inspection of time-sequences. The numbers of mitotic events were scored and the time spent in mitosis: from nuclear envelope breakdown to anaphase onset, and defects in mitosis as followed: misalignment of chromosomes or chromosomes delayed at spindle poles; lagging chromosomes; multipolar spindle and were estimated from individual cells form independent experiments. For TIRFM, cells were plated on gelatine- or fibronectine-coated glass dishes (MatTek Corporation, USA). Images were acquired using a Nikon Ti microscope equipped with a laser TIRF system controlled by the software NIS-Elements AR, a Apo TIRF 60x Oil DIC N2 objective and an Andor DU-897 X-7889 camera. The volocity software version 6.0 (Quorum Technologies) and Image J 1.48v (National Institute of Health) were used for processing on entire images before cropping to emphasize the main point of the image. Processing was limited to background subtraction, brightness/contrast adjustment and deconvolution. For experiments examining (a) the time spent in mitosis, (b) cells undergoing defective mitosis, or (c) incidence of multinucleation, the mean values of individual experiments were analyzed using the Mann-Whitney test, which is a nonparametric test that compares two unpaired groups of ordinal or numerical variables. For experiments examining (a) mitotic stages, (b) spindle angle *a*, or (c) for depletion-rescue experiments, data were analyzed using the Fisher's exact test, which is used for small sample sizes with nominal/categorical variables. Statistical calculations were performed using Prism 6.0 (GraphPad Software) statistical software.

## Supporting Information

S1 Fig Cells depleted of BAG3 show abnormal mitotic progression and nuclear abnormalities. (A) Representative mitotic pedigrees of HeLa-GFP-H2B transfected with control siRNA or Bag3 siRNA. Cells were imaged starting at 40 h after transfection for a 48 h-time period at 10 min-intervals. Each row represents the mitotic pedigree of a daughter cell, and rows are organized by families. The genealogy of cells was tracked to recapitulate their mitotic histories and to estimate the time spent in mitosis and mitotic defects; related to Fig 2B and 2C. (B, C) Western blot showing reduced levels of BAG3 in MCF7 cells treated with BAG3-specific siRNAs. The graph shows the incidence of multinucleation, which was estimated 72 h to 96 h after transfection from 2 independent experiments; n>500 cells.
(TIF)

S2 Fig Representative epifluorescence images of HeLa-RFP-H2B cells that have been transduced with recombinant adenoviruses driving the expression of BAG3-GFP proteins compared to GFP alone. Localization was analyzed 20 h post-infection; Bars, 20 μm.
(TIF)

S3 Fig Mutant BAG3 (R480A) is unable to restore aggresome-targeting of ubiquitinated protein aggregates in BAG3-depleted cells. Knockdown-rescue experiments were performed according to the protocol described in Fig 4 in HeLa-RFP-H2B cells submitted to a proteasomal stress that triggers aggresome-targeting of ubiquitinated proteins in a BAG3-dependent way (MG132 5 μM, 16 h). Deconvolved fluorescence images show representative distributions of proteins stained with anti-ubiquitin that accumulated to a perinuclear aggresome in cells treated with control siRNA (marked by arrowheads), but remained in small cytoplasmic aggregates in cells depleted of BAG3. Please note that introduction of BAG3-GFP, but not BAG3 (R480A)-GFP or GFP alone, could restore aggresome formation in BAG3-depleted cells. Bar, 10 μm.
(TIF)

S4 Fig **(A, B) HeLa-RFP-H2B cells were synchronized in mitosis by a double Thymidine-block followed by a 7 h-release period and were processed for staining of kinetochores (CREST staining), spindle microtubules (a-tubulin staining) and Hoechst.** Graph depicts the percentages of cells at different stages of mitosis upon treatment with HSPB8-specific siRNAs (A) or p62-specific siRNA (B); (A) siCTL, n = 610 cells; siBAG3_3, n = 306; siHSPB8_3, n = 181 cells; siHSPB8_2; n = 308 cells; (B) siCTL, n = 319 cells; siBAG3_3, n = 306 cells; sip62_2, n = 118 cells. (C) Deconvolved image stacks of R0-3306-treated HeLa cells infected with Ad-BAG3-GFP and Ad-RFP; red arrows designate a defined spherical perinuclear space enriched in BAG3-GFP, but not in RFP, in G2-arrested cells. (D) Western blots of total lysates prepared from nocodazole-arrested HeLa-RFP-H2B cells that have been transduced with the indicated Ad-BAG3-GFP vectors alone or with Ad-HSPB8-RFP, showing partial supershift of the BAG3 (IPV)-GFP.
(TIF)

S5 Fig Retraction fiber distribution is not significantly affected in cells depleted of BAG1 or BAG2, unlike cells depleted of BAG3. (A) Western blots of extracts of HeLa cells showing specific depletion of either BAG1, BAG2 or BAG3, after transfection of the indicated siRNAs (75 nM; 48 h); anti-BAG1 antibody recognized 3 BAG1 isoforms in HeLa cells. GAPDH levels: loading control. (B) Western blots of extracts prepared from asynchronous or nocodazole-arrested mitotic HeLa-RFP-H2B cells (40 ng/ml, 16 h). (C) Representative TIRFM images from HeLa cells at metaphase transfected with the indicated siRNAs for 48 h and transduced with BacMam-RFP-actin. Please note that unlike cells treated with BAG3-specific siRNA, cells treated with BAG1- or BAG2-specific siRNA presented numerous actin dots showing a circumferential distribution like those found in control cells, which are emphasized in enlarged views of the boxed regions and designated by red arrows. (D) Deconvolved confocal images of siRNA-treated HeLa-RFP-H2B cells transduced with Ad-LifeAct-GFP and BacMam-GFP-a-tubulin. Cells were synchronized by the double Thymidine-block method and imaged 48 h after transfection. Bars, 10 μm.
(TIF)

S6 Fig **(A) Graph depicting spindle microtubule intensity in HeLa-RFP-H2B cells treated with control siRNA compared to BAG3-specific siRNA; means of two independent experiment +/- SE are indicated.** siRNA-treated cells were synchronized with a double Thymidine block and processed for a-tubulin, cytochrome c and Hoechst staining. K-fiber intensities were calculated from confocal stacks covering the whole spindle in individual cells and normalized to cytochrome c staining in each cell (represented by individual dots) using the Volocity software. (B) Representative confocal image stacks of HeLa-RFP-H2B cells treated with the indicated siRNA, showing a-tubulin, g-tubulin, CREST and Hoechst staining. Please note that cells depleted of BAG3 showing abnormal chromosome congression (designate by arrows) did not exhibit major abnormalities in astral microtubules; Bars, 10 μm.
(TIF)

S1 Movie Related to Fig 3B—Spindle dynamics in control HeLa cells. Video microscopy of a representative HeLa cells transfected with control siRNA (siCtl) expressing low levels of RFP-a-tubulin and GFP-actin. Mitotic cells were imaged for a 1 h-period at ~1.5 min intervals using a Perkin Elmer UltraVIEW Spinning Disc Confocal and 40x air 0.75NA objective; single plane images are displayed at 7 frames/sec.
(MOV)

S2 Movie Related to Fig 3B—BAG3 depletion impairs mitotic spindle dynamics. Video microscopy of a representative siBAG3-treated HeLa cells expressing RFP-a-tubulin and GFP-actin, which show characteristic defects in mitotic spindle dynamics upon depletion of BAG3 (siBAG3). Mitotic cells were imaged for a 1 h-period at ~1.5 min intervals using a Perkin Elmer UltraVIEW Spinning Disc Confocal and 40x air 0.75NA objective; single plane images are displayed at 7 frames/sec.
(MOV)

S3 Movie Related to Fig 3B—BAG3 depletion impairs mitotic spindle dynamics. Video microscopy of a representative siBAG3-treated HeLa cells expressing RFP-a-tubulin and GFP-actin, which show characteristic mitotic spindle rocking upon depletion of BAG3 (siBAG3). Mitotic cells were imaged for a 1 h-period at ~1.5 min intervals using a Perkin Elmer UltraVIEW Spinning Disc Confocal and 40x air 0.75NA objective; single plane images are displayed at 7 frames/sec.
(MOV)

S4 Movie Related to Fig 4F—Spindle behavior upon introduction of GFP in BAG3-depleted cells. Video microscopy of a representative HeLa-RFP-H2B cells expressing low levels RFP-a-tubulin, which have been processed for knockdown-rescue. Mitotic cells expressing comparable levels of GFP alone were imaged for a 1 h to 1.5 h-time periods at ~1.5 min intervals using a Perkin Elmer UltraVIEW Spinning Disc Confocal and 40x air 0.75NA objective; single plane images are displayed at 7 frames/sec.
(MOV)

S5 Movie Related to Fig 4E and 4F—Spindle behavior upon introduction of wild type BAG3-GFP in BAG3-depleted cells. Video microscopy of a representative HeLa-RFP-H2B cells expressing low levels RFP-a-tubulin, which have been processed for knockdown-rescue. Mitotic cells expressing comparable levels of Bag3-GFP and variable expression of RFP-H2B were imaged for a 1 h to 1.5 h-time periods at ~1.5 min intervals using a Perkin Elmer UltraVIEW Spinning Disc Confocal and 40x air 0.75NA objective; single plane images are displayed at 7 frames/sec.
(MOV)

S6 Movie Related to Fig 4E and 4F— Mitotic spindle phenotype upon introduction of BAG3-GFP (IPV) unable to bind to HSPB8 in BAG3-depleted cells. Video microscopy of a representative HeLa-RFP-H2B cells expressing low levels RFP-a-tubulin, which have been processed for knockdown-rescue. Mitotic cells expressing comparable levels of Bag3-GFP (IPV) and variable expression of RFP-H2B were imaged for a 1 h to 1.5 h-time periods at ~1.5 min intervals using a Perkin Elmer UltraVIEW Spinning Disc Confocal and 40x air 0.75NA objective; single plane images are displayed at 7 frames/sec.
(MOV)

S7 Movie Related to Fig 4G—Mitotic spindle phenotype upon introduction of BAG3-GFP (R480A) unable to bind to HSC70/HSP70 in BAG3-depleted cells. Video microscopy of a representative HeLa-RFP-H2B cells expressing low levels RFP-a-tubulin, which have been processed for knockdown-rescue. Mitotic cells expressing comparable levels of Bag3-GFP (R480A) and variable expression of RFP-H2B were imaged for a 1 h to 1.5 h-time periods at ~1.5 min intervals using a Perkin Elmer UltraVIEW Spinning Disc Confocal and 40x air 0.75NA objective; single plane images are displayed at 7 frames/sec.
(MOV)

S8 Movie Related to Fig 4G—Mitotic spindle phenotype upon introduction of GFP in control cells. Video microscopy of a representative HeLa-RFP-H2B cells expressing low levels RFP-a-tubulin, which have been adenofected with sicontrol and GFP alone. Mitotic cells expressing comparable levels GFP alone and variable expression of RFP-H2B were imaged for a 1 h to 1.5 h-time periods at ~1.5 min intervals using a Perkin Elmer UltraVIEW Spinning Disc Confocal and 40x air 0.75NA objective; single plane images are displayed at 7 frames/sec.
(MOV)

S9 Movie Related to Fig 5B—Video microscopy of a representative mitotic HeLa cells transfected with siCtl, showing normal spindle behavior. Mitotic cells expressing low levels of GFP-a-tubulin were imaged for a 1h-time period at 1.5 min intervals; single plane images are displayed at 7 frames/sec.
(MOV)

S10 Movie Related to Fig 5B—Cells depleted of HSPB8 undergo abnormal rocking of the mitotic spindle. Video microscopy of a representative mitotic HeLa cells transfected with HSPB8-specific siRNA, showing abnormal spindle motility. Mitotic cells expressing low levels of GFP-a-tubulin were imaged for a 1h-time period at 1.5 min intervals using a Perkin Elmer UltraVIEW Spinning Disc Confocal and 40x air 0.75NA objective; single plane images are displayed at 7 frames/sec.
(MOV)

S11 Movie Related to Fig 5B—Cells depleted of HSPB8 undergo abnormal rocking of the mitotic spindle. Video microscopy of a representative mitotic HeLa cells transfected with HSPB8-specific siRNA, showing abnormal spindle motility. Mitotic cells expressing low levels of GFP-a-tubulin were imaged for a 1h-time period at 1.5 min intervals using a Perkin Elmer UltraVIEW Spinning Disc Confocal and 40x air 0.75NA objective; single plane images are displayed at 7 frames/sec.
(MOV)

S12 Movie Related to Fig 6B—Video microscopy of a representative mitotic HeLa cells transfected with siCtl expressing low levels of GFP-a-tubulin and RFP-actin that was imaged for a 1h-time period at 1.5 min intervals; single plane images are displayed at 7 frames/sec. (MOV)

S13 Movie Related to Fig 6B—Depletion of p62 mimics the BAG3-HSPB8-dependent phenotype on spindle dynamics. Video microscopy of a representative mitotic HeLa cells transfected with sip62, showing abnormal spindle rocking upon depletion of p62. Mitotic cells expressing low levels of GFP-a-tubulin and RFP-actin were imaged for a 1h-time period at 1.5 min intervals using a Perkin Elmer UltraVIEW Spinning Disc Confocal and 40x air 0.75NA objective; single plane images are displayed at 7 frames/sec.
(MOV)

S14 Movie Related to Fig 7D—Mitotic cortex stability in control cells. Video microscopy of a representative HeLa-RFP-H2B cell transfected with siCtl and transduced with BacMam-GFP-a-tubulin and RFP-actin; cells were imaged for a 1 h-time period at ~1.5 min intervals using a Perkin Elmer UltraVIEW Spinning Disc Confocal and 40x air 0.75NA objective and single plane images are displayed at 7 frames/sec. Please note that RFP-H2B expression is variable in this cell line.
(MOV)

S15 Movie Related to Fig 7D—siBAG3-treated cells show spectacular blebbing of the mitotic cortex along with displacement of the mitotic spindle pushed into the bleb. Video microscopy of a representative HeLa-RFP-H2B cell transfected with siBAG3 and transduced with BacMam-GFP-a-tubulin and RFP-actin; cells were imaged for a 1 h-time period at ~1.5 min intervals using a Perkin Elmer UltraVIEW Spinning Disc Confocal and 40x air 0.75NA objective and single plane images are displayed at 7 frames/sec. Please note that RFP-H2B expression is variable in this cell line.
(MOV)

S16 Movie Related to Fig 7D—Cells transfected with sip62 show dramatic blebbing of the mitotic cortex associated with mitotic spindle motility. Video microscopy of a representative HeLa-RFP-H2B cell transfected with sip62 and transduced with BacMam-GFP-a-tubulin and RFP-actin; cells were imaged for a 1 h-time period at ~1.5 min intervals using a Perkin Elmer UltraVIEW Spinning Disc Confocal and 40x air 0.75NA objective and single plane images are displayed at 7 frames/sec. Please note that RFP-H2B expression is variable in this cell line.
(MOV)

S1 Dataset Individual data point values from analyses of the complete time-lapse images. This dataset contains ten spreadsheets summarizing the data from complete time-lapse images. The spreadsheets “Fig 2B and 2C” list individual data point values from time-lapse analyses of unsynchronized HeLa-GFPH2B cells. They report mitotic pedigrees for daughter cells called by their family ID number *xyz*, where *x* is family number of the mother cell, *y* is daughter cell number on first division and *z* is daughter cell number on second division, etc… The time spent in mitosis and cytokinesis/interphase (in min) and the numbers of normal (highlighted in grey) and defective (highlighted in red) mitotic events according to the criteria listed in Fig 2 legend are listed for individual cells. The spreadsheets related to Figs 3C, 5C and 6C present cells from individual time-lapse experiments (Exp_1 to Exp_6) with their ID (field and position), mitotic progression (delineated by time-sequence intervals *Tx-y* min), and mitotic phenotype defined as spindle rocking (yellow color), stalled in mitosis +/- spindle rocking (blue color), or others (chromosome misalignment or multipolar spindle; red color). The spreadsheet related to Fig 3E presents the individual data point values for cell spindle a angle from independent experiments (Exp). The spreadsheets related to Fig 4F and 4G list cells from individual depletion-rescue experiments with their ID, mitotic progression (defined by time-sequence intervals *Tx-y* min) and mitotic phenotype: either normal highlighted in grey or defective highlighted in red (defined as spindle rocking, stalled in mitosis +/- spindle rocking, or chromosome misalignment). The spreadsheet related to Fig 7E reports all cells from individual experiments (Exp) with their ID, mitotic progression (defined by time-sequence intervals *Tx-y* min) and mitotic phenotype defined as normal (in grey), blebbing (in blue), rocking and blebbing (in yellow) or other mitotic defects (in red; mitotic delay, multipolar spindle or chromosome misalignment). The spreadsheet related to Fig 8C lists individual cells transfected with siCtl or siBAG3, that have been treated with Concanavilin A (CON A) or the vehicle for a short time-period before imaging, with their ID, mitotic progression (defined by time-sequence intervals *Tx-y* min) and mitotic phenotype: either normal (in grey) or defective (in red; defined as spindle rocking, mitotic arrest +/- rocking, cortex blebbing and/or chromosome misalignment).
(XLSX)
