# Supplementary figures and images for: A Role for the Chaperone Complex BAG3-HSPB8 in Actin Dynamics, Spindle Orientation and Proper Chromosome Segregation during Mitosis
Source: PLoS Genet. 2015 Oct 23;11(10):e1005582. doi: 10.1371/journal.pgen.1005582 (PMC4619738; doi:10.1371/journal.pgen.1005582)

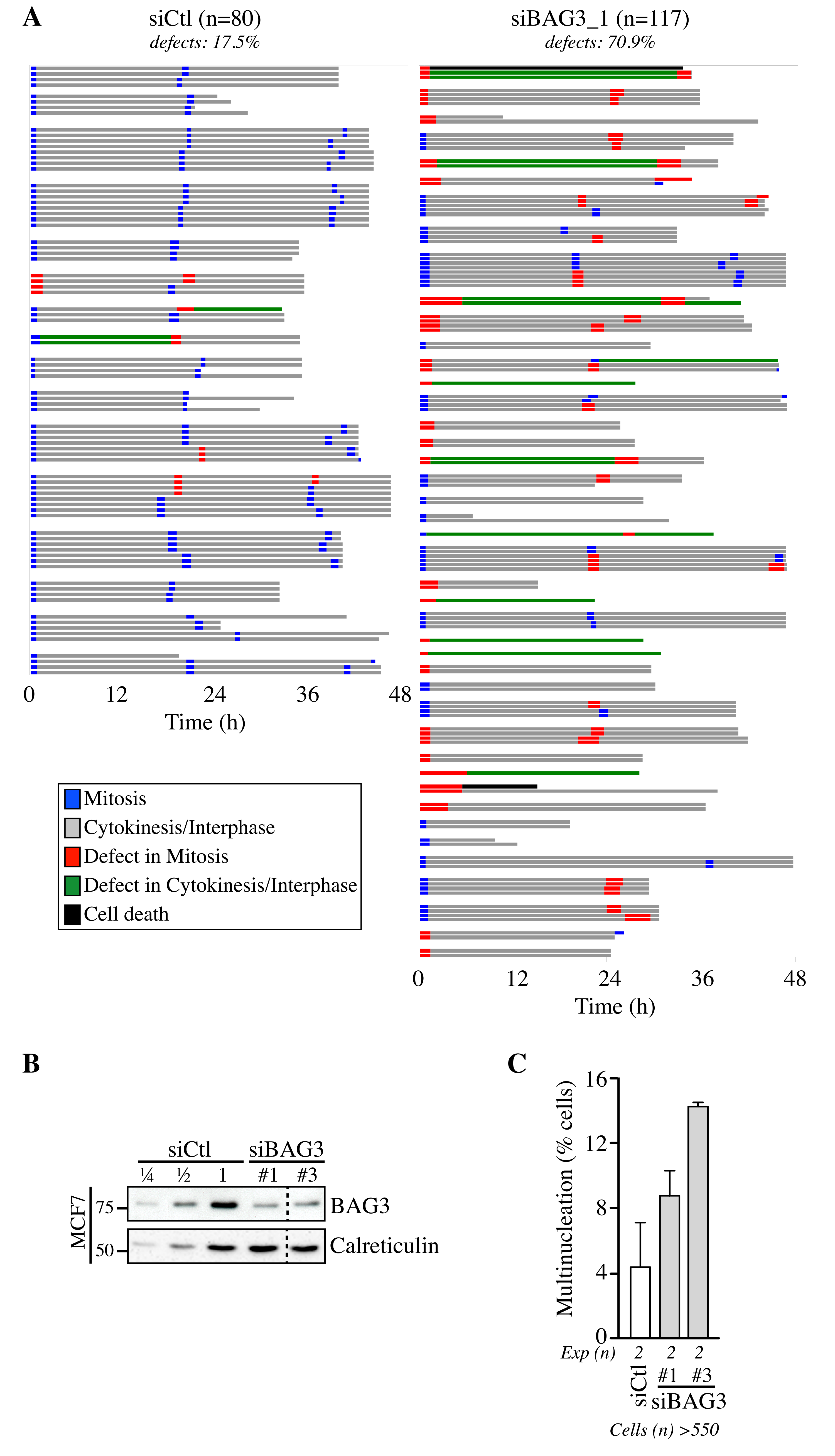

Supplement: S1 Fig — (A) Representative mitotic pedigrees of HeLa-GFP-H2B transfected with control siRNA or Bag3 siRNA. Cells were imaged starting at 40 h after transfection for a 48 h-time period at 10 min-intervals. Each row represents the mitotic pedigree of a daughter cell, and rows are organized by families. The genealogy of cells was tracked to recapitulate their mitotic histories and to estimate the time spent in mitosis and mitotic defects; related to Fig 2B and 2C. (B, C) Western blot showing reduced levels of BAG3 in MCF7 cells treated with BAG3-specific siRNAs. The graph shows the incidence of multinucleation, which was estimated 72 h to 96 h after transfection from 2 independent experiments; n>500 cells. (TIF) [file pgen.1005582.s001.tif]

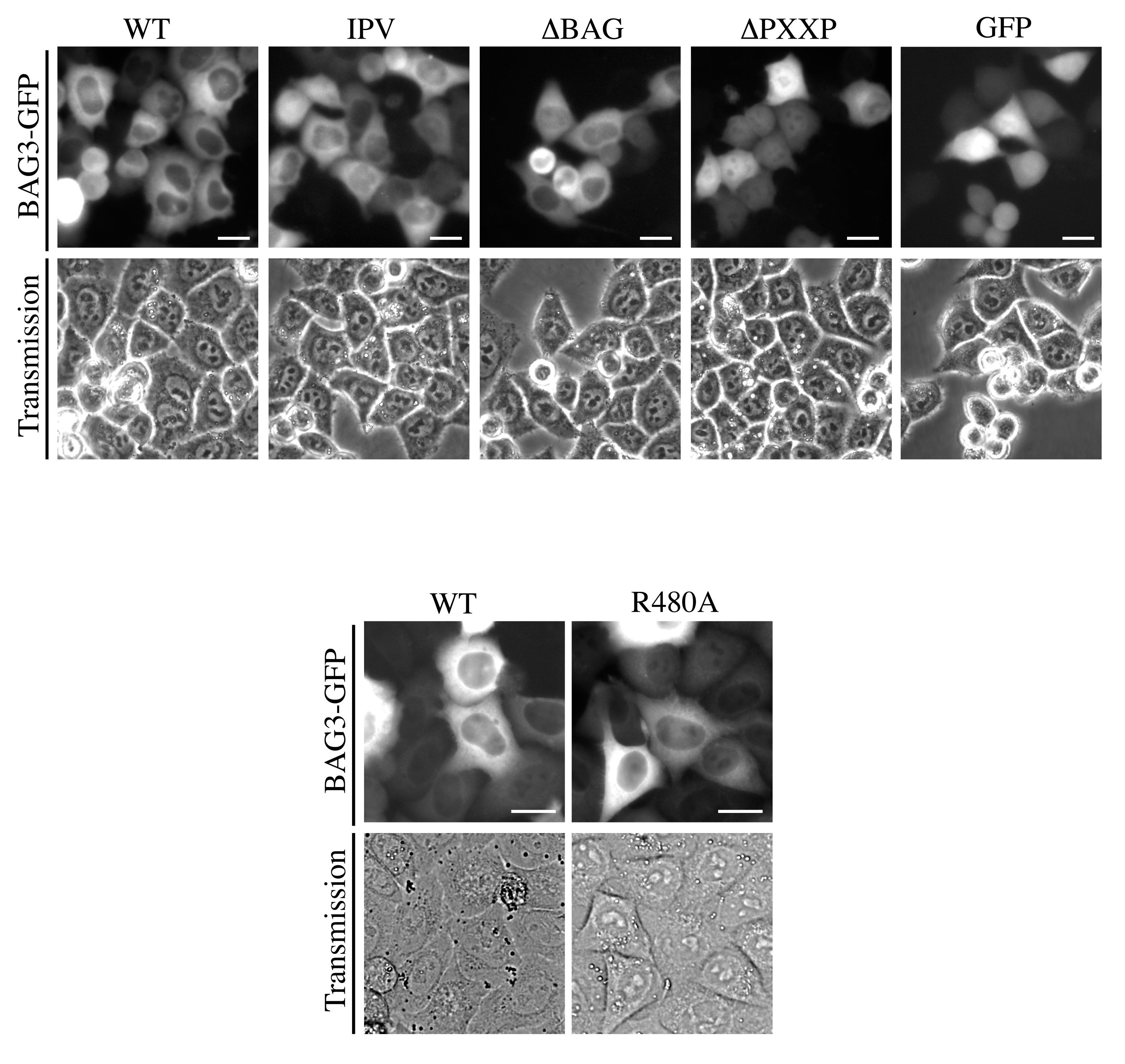

Supplement: S2 Fig — Localization was analyzed 20 h post-infection; Bars, 20 μm. (TIF) [file pgen.1005582.s002.tif]

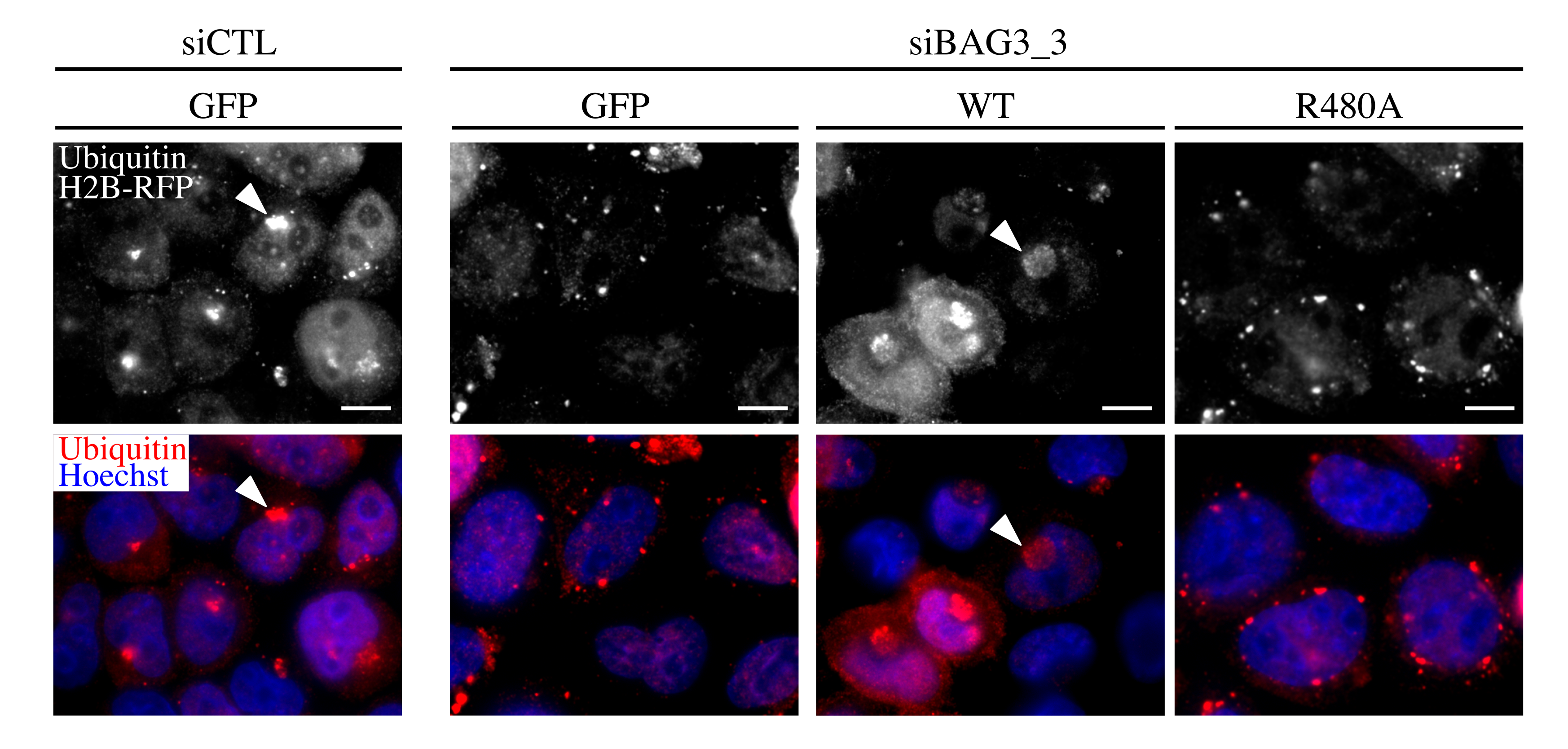

Supplement: S3 Fig — Knockdown-rescue experiments were performed according to the protocol described in Fig 4 in HeLa-RFP-H2B cells submitted to a proteasomal stress that triggers aggresome-targeting of ubiquitinated proteins in a BAG3-dependent way (MG132 5 μM, 16 h). Deconvolved fluorescence images show representative distributions of proteins stained with anti-ubiquitin that accumulated to a perinuclear aggresome in cells treated with control siRNA (marked by arrowheads), but remained in small cytoplasmic aggregates in cells depleted of BAG3. Please note that introduction of BAG3-GFP, but not BAG3 (R480A)-GFP or GFP alone, could restore aggresome formation in BAG3-depleted cells. Bar, 10 μm. (TIF) [file pgen.1005582.s003.tif]

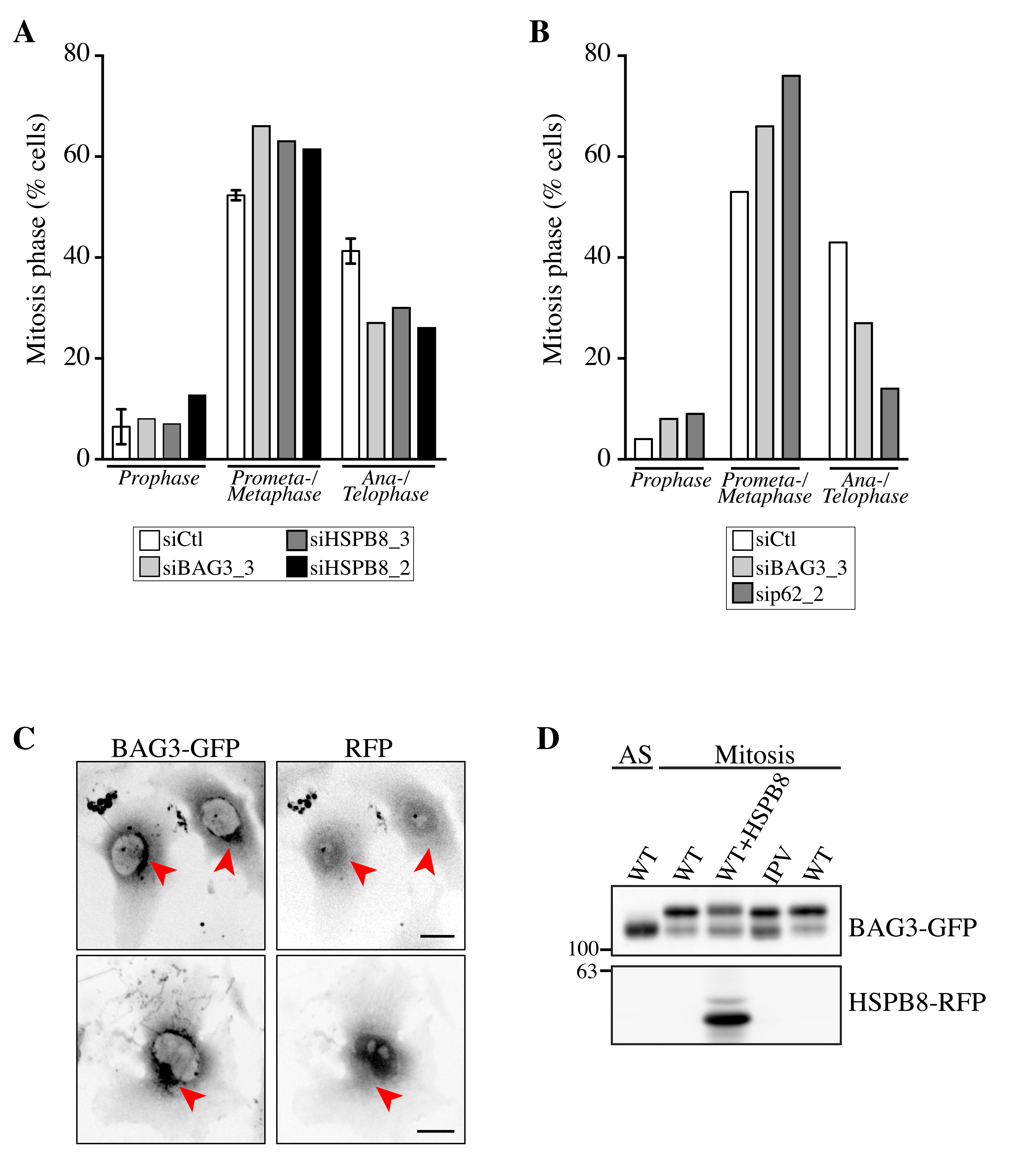

Supplement: S4 Fig — (A, B) HeLa-RFP-H2B cells were synchronized in mitosis by a double Thymidine-block followed by a 7 h-release period and were processed for staining of kinetochores (CREST staining), spindle microtubules (a-tubulin staining) and Hoechst. Graph depicts the percentages of cells at different stages of mitosis upon treatment with HSPB8-specific siRNAs (A) or p62-specific siRNA (B); (A) siCTL, n = 610 cells; siBAG3_3, n = 306; siHSPB8_3, n = 181 cells; siHSPB8_2; n = 308 cells; (B) siCTL, n = 319 cells; siBAG3_3, n = 306 cells; sip62_2, n = 118 cells. (C) Deconvolved image stacks of R0-3306-treated HeLa cells infected with Ad-BAG3-GFP and Ad-RFP; red arrows designate a defined spherical perinuclear space enriched in BAG3-GFP, but not in RFP, in G2-arrested cells. (D) Western blots of total lysates prepared from nocodazole-arrested HeLa-RFP-H2B cells that have been transduced with the indicated Ad-BAG3-GFP vectors alone or with Ad-HSPB8-RFP, showing partial supershift of the BAG3 (IPV)-GFP. (TIF) [file pgen.1005582.s004.tif]

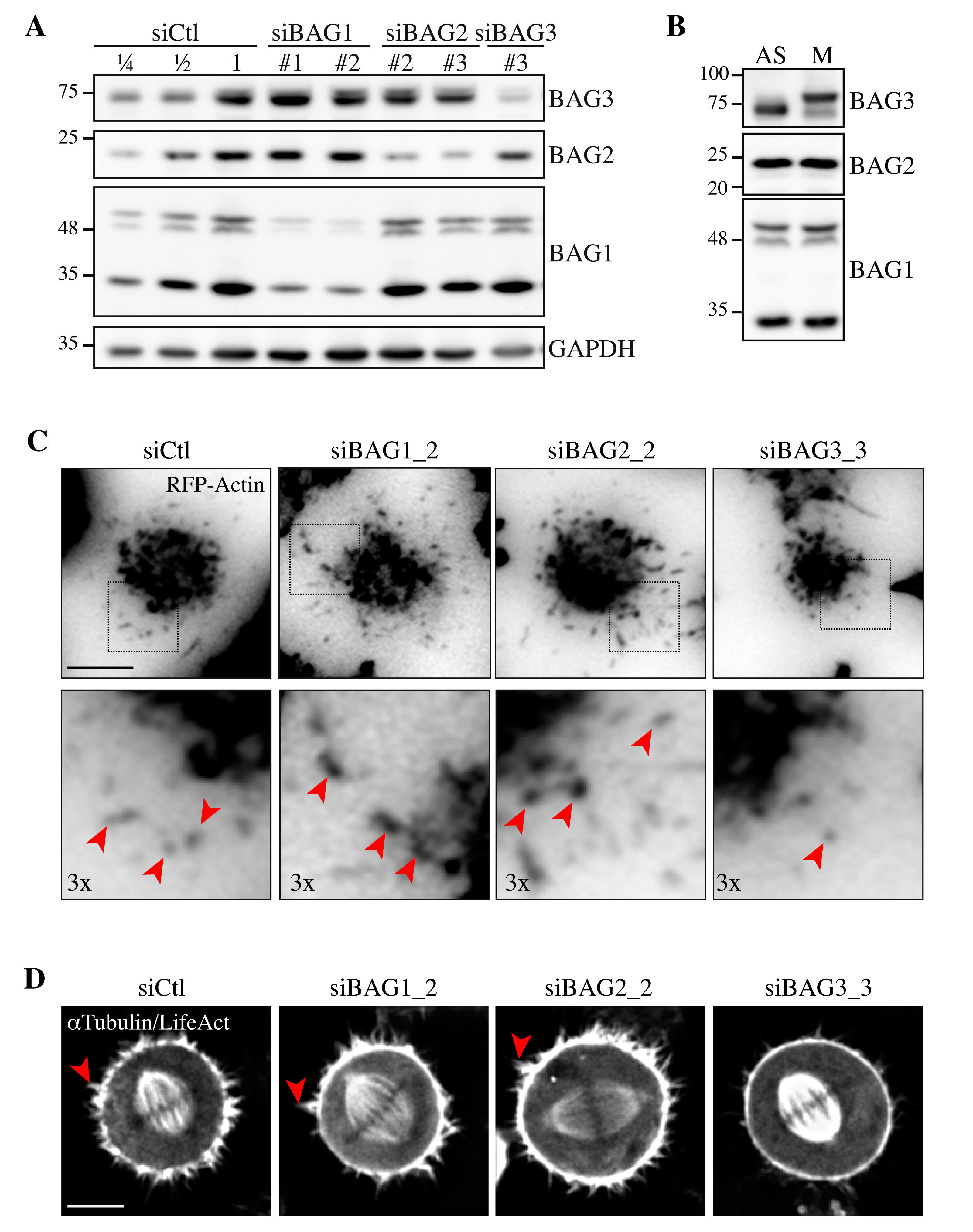

Supplement: S5 Fig — (A) Western blots of extracts of HeLa cells showing specific depletion of either BAG1, BAG2 or BAG3, after transfection of the indicated siRNAs (75 nM; 48 h); anti-BAG1 antibody recognized 3 BAG1 isoforms in HeLa cells. GAPDH levels: loading control. (B) Western blots of extracts prepared from asynchronous or nocodazole-arrested mitotic HeLa-RFP-H2B cells (40 ng/ml, 16 h). (C) Representative TIRFM images from HeLa cells at metaphase transfected with the indicated siRNAs for 48 h and transduced with BacMam-RFP-actin. Please note that unlike cells treated with BAG3-specific siRNA, cells treated with BAG1- or BAG2-specific siRNA presented numerous actin dots showing a circumferential distribution like those found in control cells, which are emphasized in enlarged views of the boxed regions and designated by red arrows. (D) Deconvolved confocal images of siRNA-treated HeLa-RFP-H2B cells transduced with Ad-LifeAct-GFP and BacMam-GFP-a-tubulin. Cells were synchronized by the double Thymidine-block method and imaged 48 h after transfection. Bars, 10 μm. (TIF) [file pgen.1005582.s005.tif]

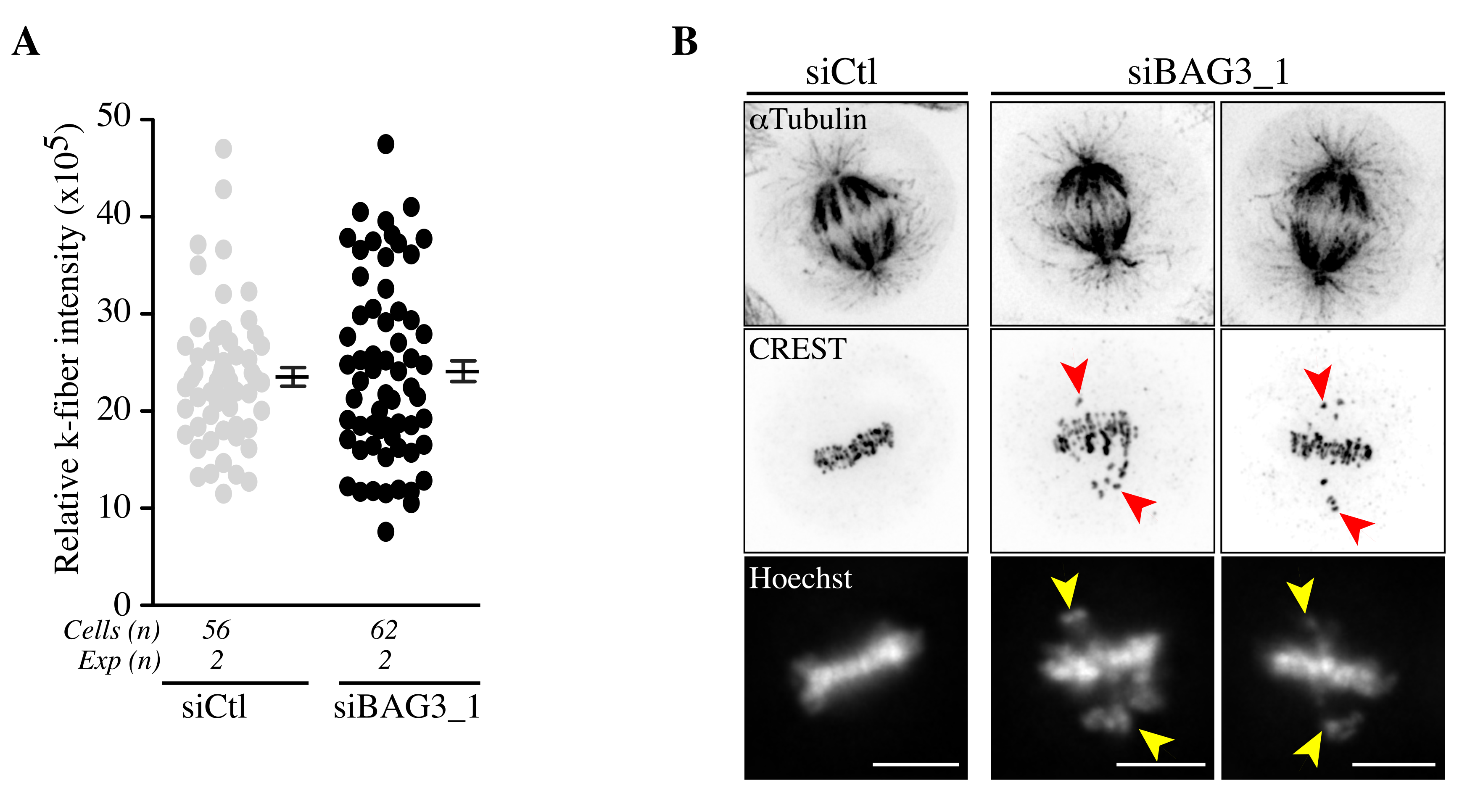

Supplement: S6 Fig — (A) Graph depicting spindle microtubule intensity in HeLa-RFP-H2B cells treated with control siRNA compared to BAG3-specific siRNA; means of two independent experiment +/- SE are indicated. siRNA-treated cells were synchronized with a double Thymidine block and processed for a-tubulin, cytochrome c and Hoechst staining. K-fiber intensities were calculated from confocal stacks covering the whole spindle in individual cells and normalized to cytochrome c staining in each cell (represented by individual dots) using the Volocity software. (B) Representative confocal image stacks of HeLa-RFP-H2B cells treated with the indicated siRNA, showing a-tubulin, g-tubulin, CREST and Hoechst staining. Please note that cells depleted of BAG3 showing abnormal chromosome congression (designate by arrows) did not exhibit major abnormalities in astral microtubules; Bars, 10 μm. (TIF) [file pgen.1005582.s006.tif]
